# disLocate: tools to rapidly quantify local intermolecular structure to assess two-dimensional order in self-assembled systems

**DOI:** 10.1038/s41598-017-18894-7

**Published:** 2018-01-24

**Authors:** Matt Bumstead, Kunyu Liang, Gregory Hanta, Lok Shu Hui, Ayse Turak

**Affiliations:** McMaster University, Department of Engineering Physics, Hamilton, L8S 4L7 Canada

## Abstract

Order classification is particularly important in photonics, optoelectronics, nanotechnology, biology, and biomedicine, as self-assembled and living systems tend to be ordered well but not perfectly. Engineering sets of experimental protocols that can accurately reproduce specific desired patterns can be a challenge when (dis)ordered outcomes look visually similar. Robust comparisons between similar samples, especially with limited data sets, need a finely tuned ensemble of accurate analysis tools. Here we introduce our numerical Mathematica package **disLocate**, a suite of tools to rapidly quantify the spatial structure of a two-dimensional dispersion of objects. The full range of tools available in **disLocate** give different insights into the quality and type of order present in a given dispersion, accessing the translational, orientational and entropic order. The utility of this package allows for researchers to extract the variation and confidence range within finite sets of data (single images) using different structure metrics to quantify local variation in disorder. Containing all metrics within one package allows for researchers to easily and rapidly extract many different parameters simultaneously, allowing robust conclusions to be drawn on the order of a given system. Quantifying the experimental trends which produce desired morphologies enables engineering of novel methods to direct self-assembly.

## Introduction

## Structural order drives properties

Order is particularly important in a wide variety of fields ranging from optics and nanotechnology to biology and biomedicine. Self-assembled nanoscale systems with low interaction, such as colloids, tend to long range, yet not perfect, order due to the competition between kinetic and thermodynamic driving forces^1^. The same is true in nature, as living systems tend to be ordered well but not perfectly^2^. Such deviations from perfect order have widespread implications including enhanced optical transmission using quasi-periodic^3^ or slightly disordered^4^ arrays of holes; controlled plasmonic response using disorder in two-dimensional arrays of nanoparticles;^5^ or two-colour bands produced by weevils on their wings from a quasi- rather than perfectly ordered photonic crystal^6^. Understanding and harnessing such effects relies on the accurate quantitative description of the extent of order in a given spatial arrangement.

The texturing of surfaces with two-dimensional dispersions of nanoscale objects (e.g. nanoparticles, micelles, quantum dots) is a particularly effective design strategy for controlling surface properties. The number, availability and spatial arrangement of such objects has been used to control metamaterial polarizatability^7^, cell growth and apopotis^8^, plasmonic enhancement^5^, cell spreading and locomotion^9,10^, transparent oxide surface work function^11^, photovoltaic conversion efficiency^12^, cell attachment on a variety of substrates^13–15^, and templated nanowire growth^16^.

**Figure 1:**
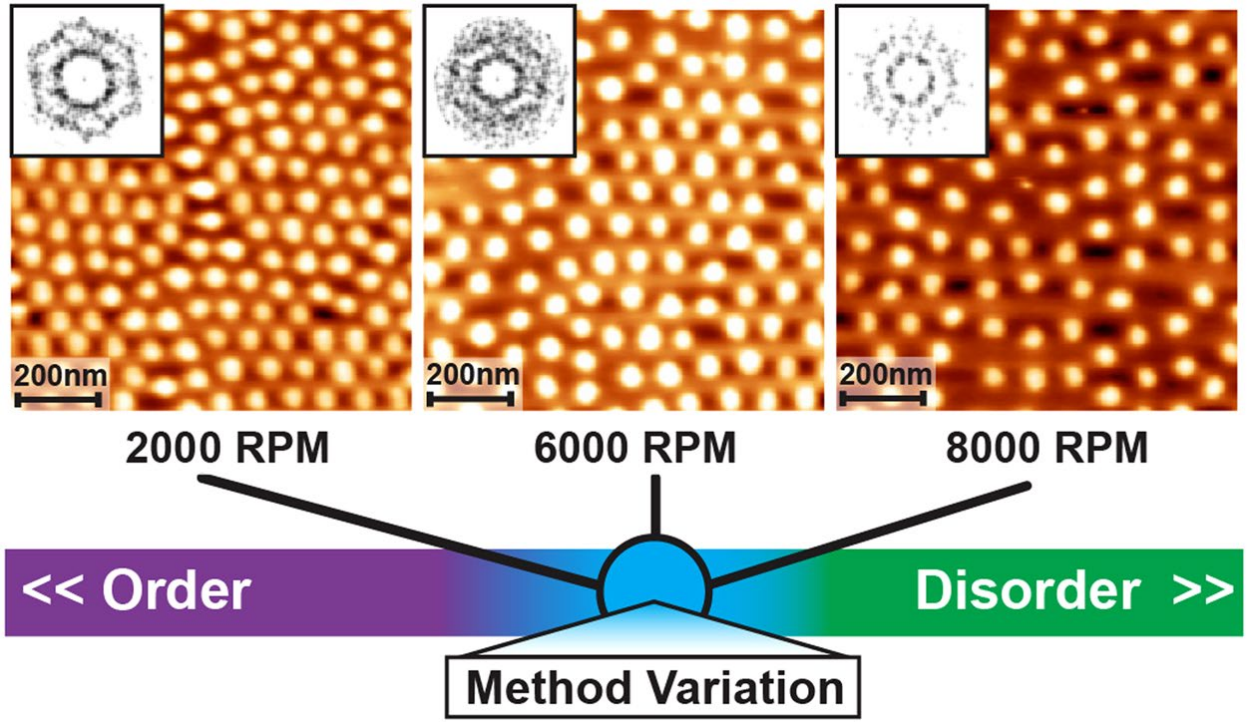
Atomic force micrographs of diblock copolymer reverse micelles (PS-b-P2VP) with varying spin-coating spin-speed (**a**) 2000 rpm, (**b**) 6000 rpm, and (**c**) 8000 rpm, showing varying spatial order. AFM images are inset with Fourier transforms of the micelle centers, showing similar planar topographies.

The ability to describe quantitatively the relative structure and ordering is highly valuable particularly to those who rely on image analysis from microscopy or other visual techniques to understand and probe experimental systems. Without tools to quantify these dispersions, observers typically rely on qualitative comparisons to draw comparisons. However, precise quantitative descriptions of the local and global arrangement are critical to consistently reproducing the desired spatial patterns to reproduce particular surface properties. As an example, Fig. 1 shows a set of AFM images of polystyrene-block-poly-2-vinylpyridine (PS-b-P2VP) diblock copolymer micelles distributed on a Si wafer surface with three different deposition approaches. Such micelle nanoreactors are typically used to form two dimensional arrays of a variety of nanoparticle materials^17–20^ to tune surface properties. It is relatively easy for observers to identify that these patterns are not in a perfectly hexagonal arrangement (highly ordered) nor are they arranged with complete randomness. The distributions of particle spacing appear to land somewhere between these two extremes.

Variations in preparation methods have an influence on the density or relative spatial distribution of such objects (see for example refs^21–23^.). Techniques such as varying the deposition spin speed are known to have this effect with diblock copolymer micelles^21,23^. In such experiments, the observable outcomes can look visually similar, as suggested by the inset Fourier transforms in Fig. 1 for these three micrographs. However, most observers can also perceive some intuitive difference in order^2^ among the three images, even if it is not immediately apparent what those differences are.

Assessing these differences requires an accurate and unbiased detection of the structural features of the objects. A common practice in image analysis is to make decisions on the relative order through a combination of user choice and computationally driven filtering, with an image analysis program such as ImageJ^24^. However, human observers often perceive order from randomness, a psychological phenomena called apophenia. Even when order does exist, human perception has difficulty in distinguishing between relatively similar ordering^2^. In order to overcome this limitation, convenient computational techniques can be used to automate research. Allowing the computer to make value judgments about experimental observations is a powerful way to save time while producing a reliable and constantly unbiased analysis. However, though computational approaches are efficient in selecting an easily measurable global mean or expectation value, the effect of variances or local descriptions still rely largely on human interpretation^25^.

Self-assembling molecular or nanoparticle systems always contain a certain amount of variation, from unavoidable measurement errors or imperceptible variations in variables at the macroscale that have visible ramifications at the micro or nanoscale. In general, rather than a perfect crystal, many self-assembling and biological systems have a polycrystalline structure – areas of high order, separated by defects and localized disorder, analogous to grain boundaries in an atomic crystal– or mesophases such as liquid crystals or plastic-crystalline systems where some of the degrees of freedom are lost^26–29^. Thus their quantification poses an issue: distinguishing any real trends can be challenging if there are limitations on sample preparation or replication due to the high cost of materials, limited quantities, or long processing times. To optimize protocols and recipes, researchers require a reliable quantitative metric to confirm the effects of changing experimental methods. Justification for a particular recipe relies on understanding how likely a system is to produce a given outcome reproducibly and how much those outcomes vary naturally. The quantitative classification of naturally occurring limits thereby provides a road-map to reproducibly produce a desired result.

In this contribution, we outline a series of tools and metrics that can be used for a fine grained understanding of both global and local spatial order patterns, within the package **disLocate** (**D**etecting **I**ntermolecular **S**tructure **Locate**d at particle positions). This provides a convenient tool, using a variety of numerical techniques, for researchers to quantify the relative disorder of a point pattern of objects and engineer desired outcomes to a higher degree of specificity. This approach goes beyond common techniques by providing access to a combination of structural metrics which estimate the amount of intermolecular order, and also introduces a way in which local fluctuations of disorder can be used as confidence range to rank protocols and experimental interventions against each other.

To outline the utility of these tools, we use an illustrative fictitious goal for spatial organization: to determine if it is possible to direct the self-assembly of our diblock copolymer micelles from solution shown in Fig. 1 into a polycrystalline hexagonal periodic arrangement with internal spacing of two micelle diameters between particles using the spin coating speed. In this example, to showcase the power of such an approach for limited data sets, we use the realistic scenario where AFM micrographs from three experiments of varying spin speed are available. This leaves the next possible experiment within three choices to achieve our goal: 1) decrease the spin speed below 2000 rpm, 2) refine the spin speed between 2000 and 8000 rpm or 3) increase the spin speed above 8000 rpm. Each of the tools available in the package will be used consecutively on the point patterns extracted from the experimental data, described in relation to a conclusion based solely on observation, contrasted against an interpretation based on the numerical metrics. Using the full range of information available in **disLocate**, we are able to distinguish between the three images with a degree of specificity that allows us to determine if it is feasible to reach our fictitious goal with this experimental procedure.

Based on the procedure described in the following sections, we were able to use **disLocate** to identify that increasing speed will increase the intermolecular spacing at the expense of the angular periodicity, and that an intermediate speed may be sufficient for our purposes. The full range of tools available in **disLocate** gave different insights into the quality and type of order present in the various samples. Using **disLocate** for spatial analysis would also allow us to quickly change experimental tactics if necessary (such as modifying solvents, temperature, or polymer) by providing an accurate trend with a limited amount of data.

### Brief overview of “disLocate”

The **disLocate** package was developed to provide a series of automated tools to quantify the varying degrees of order that can exist within a given spatial distribution pattern. Though highly ordered patterns are relatively easy to classify, disordered or nearly ordered states as exist in a variety of biological and nanoscale systems are much more difficult to distinguish effectively.

Additionally, the trend in nanoscale research is for higher resolution images of smaller surface features. This limits the number of objects observed, which truncates the information each image has and decreases the effectiveness of a statistical approach. The resulting loss of information from limited long range order curtails the use of standard numerical techniques. It is possible for researchers to misclassify and assign a false positive to the reproducibility of their experiments by simply adapting generally used metrics designed for systems of many objects. This issue can be avoided by adopting a new set of techniques which consist of structural metrics that can distinguish local differences in the observable structural order rather than solely relying on global averages of samples (though those are also available directly from the package if so desired).

Order on the local scale can be sub-categorized into three distinct types:

- Translational order occurs when every particle in the system has an exact position that repeats at a specific distance, defined by a specific translation period.
- Entropic order is the amount of free volume (sections unoccupied by particle mass) encompassed by the system, where highly ordered systems have the lowest possible free volume (maximum density).
- Angular order relies on relative arc-separation of the “bonds” between a particle and its neighbours.

The expectation value for complete periodicity occurs when neighbours for any given particle have the same arc symmetry as well as being equidistant, with maximized covering area due to the equivalent position of each particle relative to all others. If any one of these types of order are not met, the system can be considered in a mesophase^28^, such as that observed for plastic crystals (limited orientational or rotational order, but long range translational order)^30^ or liquid crystals (limited translational order but long range angular order)^31^.

**Figure 2:**
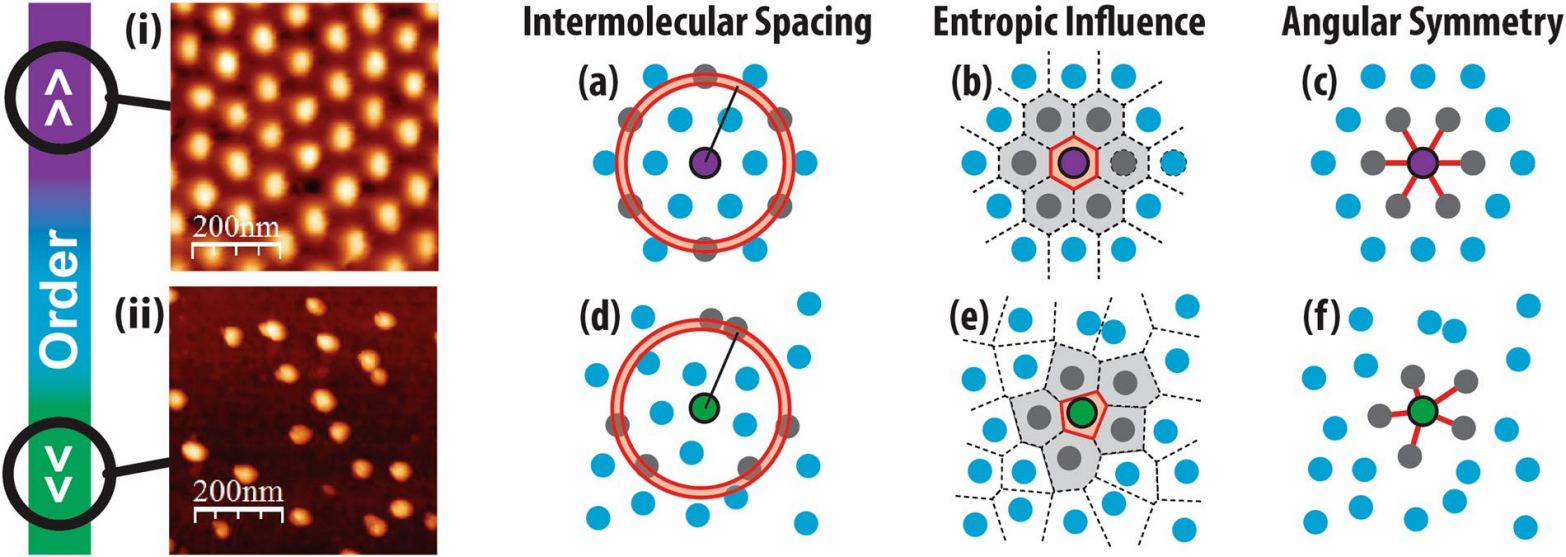
(left) Self-assembled morphologies land somewhere between highly ordered systems (top) and ones with very low density (bottom). Two key factors in classifying disorder are simultaneously in competition with each other: limitations on perception misguides observers into seeing patterns in randomness while the imprecise ability to distinguish between similar patterns misses subtle differences. To remove unwanted bias, numerical order metrics are utilized to characterize the morphology located at particle positions: pair correlation function (**a** and **d**), Voronoi tessellations (**b** and **e**), and the bond order parameter (**c** and **f**).

Each of these types of order requires a different tool to extract the desired information, as shown schematically in Fig. 2. Positional order can be described probxabilistically using the pair correlation function^32^ (Fig. 2(a) and (d)). This tool counts the number of objects within a small shell at a distance away from a central particle and averaged over all particles in the system. Local free volume and the complementary metric, covering area^33^, can be calculated by partitioning the substrate into a Voronoi tessellation around each individual particle (Fig. 2(b) and (e))^34^. This routine draws a perpendicular line at the midpoint along the line-of-sight vector connecting nearest neighbours around every particle. Angular order can be calculated by using the bond order parameter^35^, which compares the angle between the central particle and its closest neighbours against a specified symmetry basis vectors (Fig. 2(c) and (f)). All of these methods have been widely used to characterize disorder, identify polycrystalline and disordered sections, and extract the probability of intermolecular spacings^36,37^.

For comprehensive analysis, all of these techniques are bundled together as a set of tools in a freely distributable Mathematica package (disLocate.m) (available in the Supporting Information) to build sets of hierarchal metrics that can distinguish morphological subtleties. Table 1 outlines specific physical parameters that describe types of structural order within planar morphologies that can be analyzed with this package. The main thread that ties these tools together is the partitioning of space into local Voronoi tessellations around each particle as a basis for critical decision making on the extent of order. The variation in each of the above metrics can be provided by the information contained within the Voronoi polygons unique to each particle. This provides the statistical basis for quantifying different dispersions with a confidence range that may be unaccessible due to the limited numbers of prepared samples. Though AFM micrographs are used in the following sections to illustrate the power of **disLocate** in classifying spatial organization, our package relies solely on the point pattern generated by the centroids of the particles. As such, it provides a valuable tool for quantitative microscopy across any length scale as shown by the representative SEM, AFM and optical micrographs in Figs S1–S3 in the Supporting Information.

**Table 1:** Summary of physical observables and the tool within **disLocate** that has the ability quantify them. The global observable refers to an invariant between different observers, either by simplicity of counting or common routines. The mean local reference utilizes the internal properties of the single data set to determine an expected mean for the physical properties. The variations are products of defining these metrics as localized parameters. It should be noted that confidence in specific metrics can be enhanced by capturing the structural variation from another one of the analysis tools, since the variation will have a correlated influence between these through the Voronoi partitions.

	Global Observable	Mean Localized Reference	Disorder Variance and Confidence
Intermolecular Spacing	Pair Correlation $g{(r)}_{{\rm{\max }}}^{{1}^{{\rm{st}}}}$	Expected Hexagonal Lattice 2*r*_*hex*_	Hexatic Mean Displacement Δ*r*
Entropic Coordination	First Neighbour Shell $g{(r)}_{{\rm{shell}}}^{{1}^{{\rm{st}}}}$	Coordination Bond Structure of symmetry $\ell$ Fig. 4	Ratio of Coordination ${N}_{(\ell )}/{N}_{{(\ell )}_{6}}$
Angular Symmetry	Hexagonal Bond Order $\langle {q}_{6}\rangle /{q}_{6}^{hex}$	$\ell$-fold Bond Order $\langle {q}_{\ell }\rangle /{q}_{\ell }^{sym}$	Deviation in ${q}_{\ell }$ from Random Δ*q*_5_ Δ*q*_7_

## Results

### It is harder to distinguish differences in positional order when experiments have similar density

When defining order, the most common feature that humans quantify is the positional structure – i.e. the relative distances between objects arranged in a particular pattern. Variations in density are typically the first noticeable feature observed between sets, before confirmation with counting of each individual object. Standard analytics such as number count (*N*), mean radius <*r*>, and the covering area (*ϕ*) are global structure metrics that describe the density of a morphology on a surface. Table SI (Supporting information) outlines the calculated global values for the micelle system extracted from the micrographs using the image analysis software ImageJ. Using this information, it is clear that the number count and covering density decrease with spin speed, but that is all the information we can extract. To extract the positional and symmetry data, a standard approach is to take the fast fourier transform (FFT) of the image^38,39^. However, systems where these features are similar, such as the micellar systems in Fig. 1, require other tools to describe the varying types of order.

The positional structure of a dispersion describes how the number density of objects behaves as a function of relative distances. One of the key measures is the average internal spacing between the centroids of objects. If the objects are evenly spaced out at specific distances by lateral translations of a repeated unit (unit cell), the periodic order can be easily understood and quickly recognized by observers. For two dimensional systems, these translations are limited to one of five Bravais lattices. For our fictitious goal to quantify and objectively distinguish the ideal experimental condition, the aim is to form a hexagonal (triangular) disperse lattice. In such a hexagonal lattice, the distances between the object centres are all of the same length, separated by an angle of 2*π*/6, such that a single object is surrounded by six equidistant neighbours. If the objects are touching (i.e. in a closed-packed array) the distance to finding another object is easily determined as twice the radius of the particle. This distance sets the minimum separation between spherical objects. As the objects are not exactly touching in our desired arrangement, the centre to centre distance can instead be described by an effective radius (*r*_*hex*_), half the distance between the object centres.

If the objects are arranged in a predictable lattice pattern (such as Fig. 2(i)), the local density is identical to the global average density of particles, and is correlated to this radius. However, if there is no correlation between positional arrangement and density (as in Fig. 2(ii)), the pattern corresponds to a condition of complete spatial randomness. In such a condition, the position of each object is unrelated to any of the neighbour positions, suggesting no interactions between objects. The mean density does not correlate to any important structural feature, and as the pattern is arbitrary, there is no expectation that any experimental intervention will be able to reliably reproduce the observed pattern.

In between these two extremes, the spatially disordered systems can be characterized by internal distances between particles which vary slightly throughout the system. This implies that the density changes at a localized scale while retaining the average global density.

**Figure 3:**
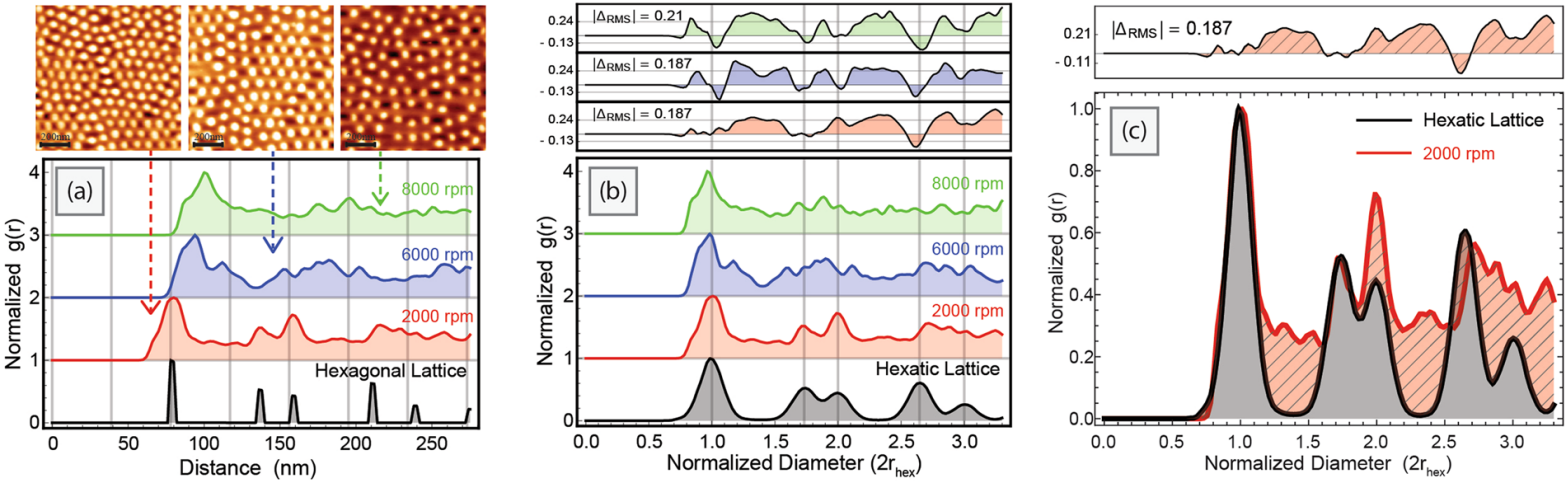
Pair correlation functions of centroids obtained from AFM images of diblock copolymer reverse micelles (PS-b-P2VP). (**a**) Measurements of objects shows that the peak positions are misaligned. This implies the average spacing between micelles changes with spin speed. (**b**) Normalizing the distance to the average spacing (2*r*_hex_) of a hexagonal lattice with the same number density (located at particle positions) collapses the distributions into a shared spatial reference frame where peak positions can easily be compared. (**c**) The differences in *g*(*r*) is shown as red thatched sections on an overlay of both functions from 2000 rpm micelle distributions and the matched hexatic lattice. The widths of these peaks correspond to the average mean displacement each particle has relative to this expected spacing. The inset above (**b**) and (**c**) shows the difference spectrum, which is the subtraction between a pair correlation function and the hexatic lattice.

The pair correlation function *g*(*r*) describes the radially averaged density as a global function of positional distance away from every particle (see Fig. 2(a) and (d) and Methods 4.4). In a perfect hexagonal lattice, this would result in delta functions with no positional variation at radial distances where neighbours are located on the unit lattice with known values^40^ (see Fig. 3(a)).

One way of quantifying the inter-object spacing is to use the maximum of the first peak of the pair correlation function, which are plotted in Fig. 3(a). These values are outlined in Table SII (Supporting Information) which shows a trend of increasing spacing as the spin speed is increased, as expected. To determine if our goal of increasing the mean spacing has been met, we require an accurate and reproducible value for this inter-object spacing. Instead of relying only on a single global value of probable spacing from the *g*(*r*), we also implement a set of routines that can extract the mean local spacing from the Voronoi tesselations. Voronoi tessellations partition the space into sections that encompass the centroids of the objects in the system. This produces localized partitions that express the maximum space each particle can potentially posses. Each individual particle will produce a unique tessellation that corresponds directly to the relative position of its neighbours, which can give information on localized preferential structural order (see also section 3.2).

This method can be used to extract the mean expected spacing $\langle 2{r}_{hex}^{vor}\rangle$ using the local density without measuring any distances between particles. The area of the Voronoi cells can additionally be used to directly calculate the effective radius 2*r*_*hex*_ that a hexagonal lattice would have to possess to have the same local Voronoi area.

This approach based on local Voronoi areas also provides a statistical distribution in the local density from which the variance in localized translational disorder can be extracted. We designate the intermolecular variance using the density located at particle positions as the lattice disorder parameter (Δ*r*). This variance information is generally difficult to extract directly from the *g*(*r*).

As seen in Table SII (Supporting information), the mean expected localized spacing, $\langle 2{r}_{hex}^{vor}\rangle$ is larger than the globally derived spacing from the effective radius or the *g*(*r*). This is a direct result of global edge artifacts. As the particles in the image represent a subunit of a larger surface, the particles on the edge have neighbours outside the image that should contribute to the calculation of density. With an unknown local area outside of edge of the image, including edge particles results in an artificially lower total particle number and density, due to the increased ratio between the number of particles and the observed area. The Voronoi tessellation reduces the error associated with particles on the image edge, by allowing for the removal of such edge particles, to more accurately define the intermolecular area located at particle positions.

Using this refined effective intermolecular spacing, we observe that increasing the spin speed to 6000 rpm achieves a value ~100 nm (see Supporting Information Table SII). As each micelle has an approximate diameter of 50 nm (Supporting Information Table SII), this spacing corresponds to our goal of achieving a sparse lattice of approximately two micelle diameters.

Having established that increasing the spin speed beyond 6000 rpm is effective in increasing the spacing, which was relatively easy to determine using only the global metrics from the pair correlation function, one now needs to establish how well the translational order was preserved under those same conditions.

One key metric for order comes from the pair correlation function: the extent or degree of order can be inferred from the number of peaks that appear — each peak corresponds to another further neighbour at the expected location. High probability of finding neighbours where the lattice translations would predict them to be, as shown by peaks in the *g*(*r*), is suggestive of order. A delta function at five times the effective radius, for example, would suggest that the order is preserved at long range. As can be seen in the rest of Fig. 3(a), none of the *g*(*r*) distributions for the micelles systems under different experimental conditions conform to a perfectly ordered hexagonal lattice at the bottom (calculated using the number density of the 2000 rpm micelle image). As the density for each image is slightly different, it is difficult to directly compare how the pair correlation functions differ, to judge changes to the ordering from the change to the spin speed.

To accurately determine the degree of order, it is necessary to separate the information regarding relative spacing of particles from their probability for translational disorder. By normalizing the real-space distances to the intermolecular effective radius (the first nearest neighbour effective radius expectation value), it is possible to evenly quantify the deviation in positional order amongst samples. Figure 3(b) outlines this normalization process, such that all functions now share the first maximum peak at the same distance. The package automatically extracts the residual difference spectrum, Δ*g*(*r*)^41^, (Fig. 3(b) top panel), which allows researchers to quickly determine the relative amount of positional fluctuation of particles relative to any reference data set. Areas above the curve indicate more probability for particles to be at this normalized distance compared to the reference. The maximum Δ*g*(*r*) difference between the reference lattice occurs when the data has complete spatial randomness (i.e. no spatial correlation) and is minimized when the data exactly matches the reference.

As the Bravais lattices are strictly defined by exact spatial translations in two dimensions, deviations necessarily mean that positional order is lost. However, with only slight disorder, the global features of near order are easily recognizable to most human viewers. It is common for researchers to use the spatial order classifications of “quasi-hexagonal”^42^ and “hexatic”^43,44^ to define systems that are close to hexagonal, often using spatial symmetry to define such a system (see section 3.3). In the pair correlation function, slight deviations of the particles in the vicinity of the global lattice positions will not change the mean particle spacings, and hence the neighbour peak positions; however, each of these peaks do experience a broadening of the peak widths to account for how the small fluctuations increase the chance for neighbours to be found at a different distance (see supporting information Fig. S4). The approach to determining the $\langle 2{r}_{hex}^{vor}\rangle$ intermolecular spacing provides a powerful tool to understand this behaviour. With the local Voronoi areas, a statistical distribution in the local density is found for which the variance in localized translational disorder can be extracted. We propose, therefore, that the pair correlation function can also be used to quantitatively distinguish differences between patterns by systematic normalization to a new lattice: the “hexatic” lattice.

Using our tool set, a “hexatic” lattice is determined through an ensemble average of a hexagonal lattice which has been modified by randomly displacing the particles from their center with a mean distance relative to a normal probability distribution. This approach can be applied to any Bravais lattice or in fact any distribution of points. The lattice disorder parameter (Δ*r*) directly relates the random intermolecular displacement to the expected lattice spacing.

A larger value of Δ*r* between one sample relative to another indicates more translational disorder (i.e. less positional correlation) by having higher spreads in the intermolecular distances. This way it provides a model to use the local density for quantifying random displacements located at particle positions. For the series of micelles deposited at different spin speeds, the lattice disorder parameter (Δ*r*) (supporting information Table SII) increases with greater spin coating speed, suggesting that peaks are broadening in the pair correlation function, as is observed.

A single overlay comparison between a simulated hexatic lattice with the disorder parameter and an experimental micelle *g*(*r*) is shown in Fig. 3(c) with the distances normalized by the expected hexagonal spacing. To quantify differences in disorder outside the first neighbours, the *g*(*r*) functions are also normalized by their maximum value (i.e. highest probability is at first neighbour diameter). The Δ*g*(*r*), pair correlation difference spectrum, again gives a snapshot view of how the experimental data deviates from the reference spectrum, in this case the slightly disordered hexatic lattice.

Hexagonal ordering has a unique feature in the *g*(*r*) that corresponds to a splitting of the second shell of neighbours into two peaks, seen in Fig. 3 for both the hexagonal and hexatic lattices. If the second neighbour peaks overlap almost completely, and there are peaks in the pair correlation function centred around 1.75 and 2 normalized diameters, the system is much more likely to have long-range global hexagonal periodic order. This feature can be used as a quick test to determine if a pattern is likely to be hexatic, rather than possess another type of order (see Fig. S5 for examples of the expected pair correlation functions for various lattices). However, this examination is not definitive, as defects, polycrystallinity and disorder can also lead to splitting in the pair correlation function peaks^45–47^. Figure 3(c) suggests that the 2000 rpm micelle data has hexatic order as indicated by large overlap between functions at both the second neighbour expected hexagonal spacings. The splitting of the second order peak exists, though the fitting is not as good for the peak centred around 2 normalized diameters, suggesting some sections that may have other spatial symmetries (square lattice, twinning defects, etc)^45–47^. The appearance of a peak close to that expected for the third nearest neighbour is also suggestive of global hexagonality, but the deviations between the experimental data and the hexatic lattice grow more pronounced as the distances increase, suggesting a large number of defects and grain boundaries. In data sets of limited particles, the pair correlation begins to fail at long distances, due to the missing information of particles who have intermolecular distances which lie outside the image. The region between the first and second neighbour shells (cross-hatched area in Fig. 3(c)) also suggests that the micelles have more disorder than a pure hexatic lattice at these distances. Overall, the comparison to the hexatic lattice for the micelle data does suggest that there is a high degree of “hexatic” character for 2000 rpm spin speed, at least to the level of the second nearest neighbour shell, but with some defects.

For spin speed as a parameter, the local fluctuations in spatial positions increase as the hexagonal lattice spacing increases, leading to less “hexatic” character. The “hexatic” lattice defined by the lattice disorder parameter for the other spin speeds is given in supporting information Fig. S6. With such a large Δ*r* for 8000 rpm, the hexatic peaks broaden to the extent that there is almost an equal likelihood of finding a particle at any distance, similar to a liquid or glass. This is also supported by the average mean square displacement from the difference spectra, Δ_*rms*_ which increases as the hexagonal lattice spacing increases, due to the relative broadening of all the *g*(*r*) peaks (Δ*g*(*r*) spectrum in Fig. 3(b)). This behaviour suggests that the spin-speed has multiple effects on the spatial order: the higher centripetal forces with increasing spin speed increases the intermolecular spacing while also increasing the amount of fluctuation between neighbours distances. This might suggest that the positional order is somewhat sacrificed when increasing the spacing using this approach. This is most evident for the highest spin speeds, which achieves the goal of increasing the spacing to twice the micelle radius, but appears to completely lose most of the positional hexagonal order. Lower spin speeds, however, retain a hexatic formation while increasing the spacing by a lower amount. Using a combined method of implying both local and global density metrics, it is possible to amplify the key influence that experimental variation has on intermolecular spacing.

### Extracting entropic force information by grouping particles with similar local bond structures

As described in section 3.1, the translational separation within point patterns is relatively easily recognized by both human and algorithmic observers if the metric is relaxed enough to allow for some small fluctuations. The variance in positional order is particularly helpful in determining the usefulness of particular experimental interventions. The lattice disorder parameter, however, only gives a global metric relating to the deviations from a perfect lattice. It does not reveal if the deviations are uniformly dispersed, if these systems are polycrystalline – systems with areas of local order separated by defects – or if they exist in a mesophase, where some type of order (translational, entropic, or angular) is not satisfied^28,48^. Though spatial disorder is fairly intuitive to classify, distinguishing between systems consisting of other types of order can be challenging for researchers.

The entropic order is related to the unoccupied areas in a given dispersion, that is, the available free volume. Necessarily, when the free volume is in the lowest possible state (i.e. maximum density), the system is in a highly ordered state. The drive to minimize the free volume, the entropic order, can therefore be harnessed to drive self-assembly, as has been observed recently for a variety of systems^29,48–52^.

Particularly, in anisotropic systems (i.e. with non-spherical shapes^48,51,53^ or asymmetrically functionalized coated spheres^52,54^), there is a driving force that aligns faceted or functionalized particles so as to maximize the system entropy by minimizing the free volume^50,53^. The so called directional entropic force^51,55^ drives systems to complex structures. The anisotropic probability distribution describing the likely positions of neighbouring particles arising from this force is analogous to that observed for chemical valence states^53^.

In chemical systems, the valence defines the coordination number of an object: i.e. the number of neighbours to which the object is bonded. In the absence of bonds, as in our micelle systems, the entropic force yields a similar “coordination number” defined through the number of nearest neighbours. The drive to maximize local entropy is therefore related to the number of nearest neighbours, even for systems without any intrinsic anisotropy.

Observers have a hard time distinguishing the extent to which the free volume is minimized in a particular system. Due to the various neighbour definitions that exist^56^, defining the neighbours correctly also poses challenges. The selection of neighbours is truly a binary selection criteria: it either fits the definition and is counted as a neighbour or it is rejected from analysis. Utilizing numerical methods allows for a more refined and accurate detection of neighbours than achievable by an observer alone. However, subtle differences in particle positions (i.e. numerical accuracy) can lead to the computer providing a neighbour list that may differ from ones provided by the qualitative decisions of an observer, leading to large differences in the classification of structures.

To achieve consistency in algorithmic classification, methodological numerical consistency should be favoured across comparators rather than variable definitions^57^ that can change depending on the sample. In our package, Voronoi tessellations are chosen as a robust method to calculate neighbours due to their invariability with respect to particle size and shape. Such an approach yields an unbiased definition for the coordination number for each object when the distance between particles is much larger than its size (low density)^56^. The coordination number is defined through the number of Voronoi cell facets which contain the particle (see Fig. 2(b) and (e)). By partitioning each particle, Voronoi tessellations can be used to accurately calculate the entropic order through the minimization of the free volume around each particle.

**Figure 4:**
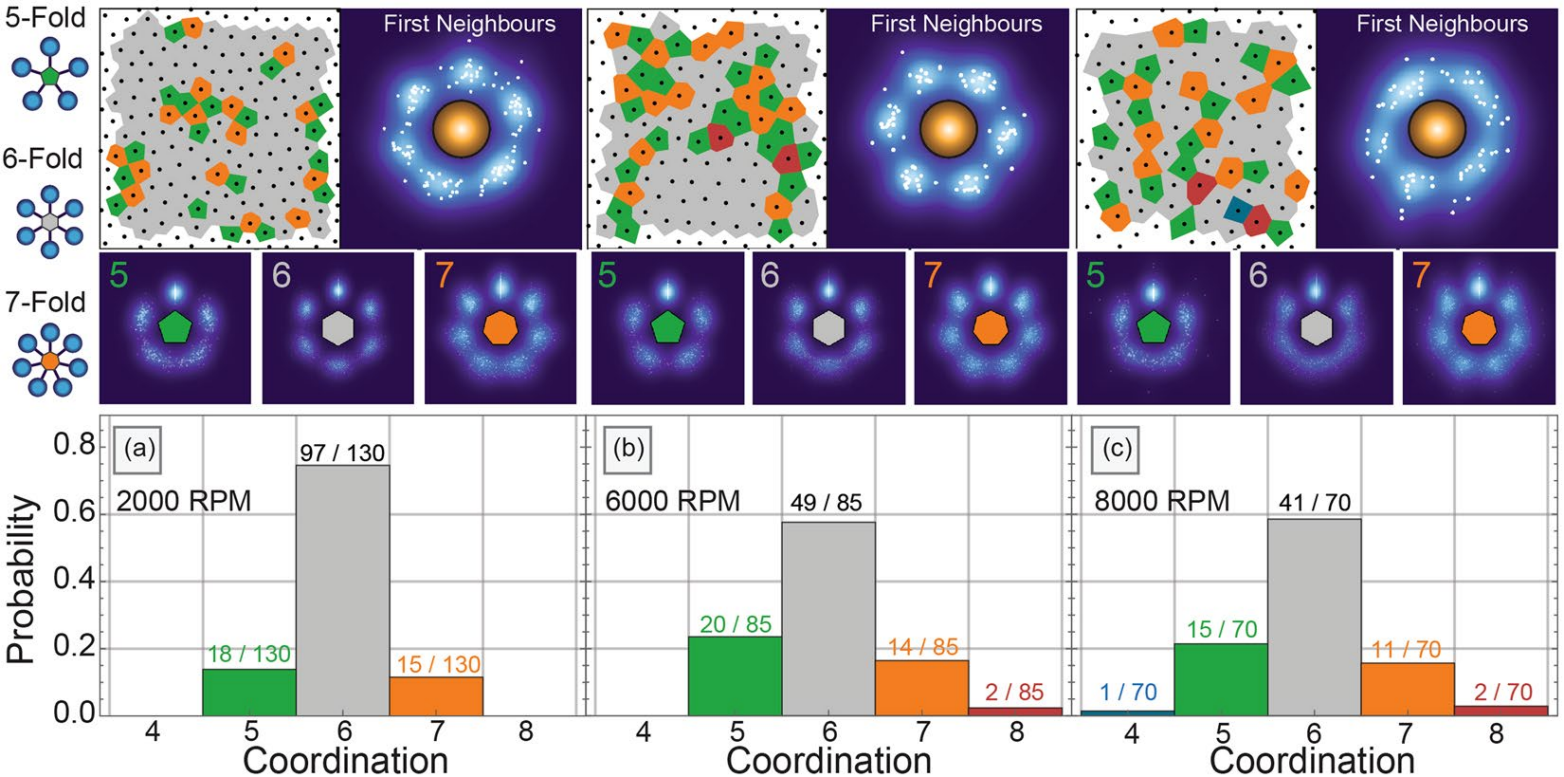
Angular classification of coordination neighbours within micelle configurations observed by AFM. Coordination numbers are calculated using the Voronoi tessellations (top panels left), given in a histogram of the probabilities for each coordination number (bottom panels). Particles are translated to have a common origin so as to build a planar probability map of first neighbour (entropic force map) in relation to the particle center (top panels right). Particles are then separated by their coordination number and remapped to observe correlation between the expected angular symmetry (shown on the far left) and the rotational order of neighbours (middle panels). Bond-structures are rotated to have a common orientation (y-axis) so as to show disordered states as blurred rings at the bottom (negative y-axis) and ordered states as sharp bright spots. Experimental spin speeds (**a**) 2000 rpm (**b**) 6000 rpm (**c**) 8000 rpm.

Fig. 4 outlines probability histograms for local coordination numbers for each spin speed of our micelle system, defined for ease of identification at the far left-hand side. Above (left hand side for each panel), the Voronoi tessellations for each image used to generate the histograms are shown. They are overlaid on a representation of the particle centroids, plotted using a colour scheme that highlights the positional arrangement of particles with similar coordination number.

**Figure 5:**
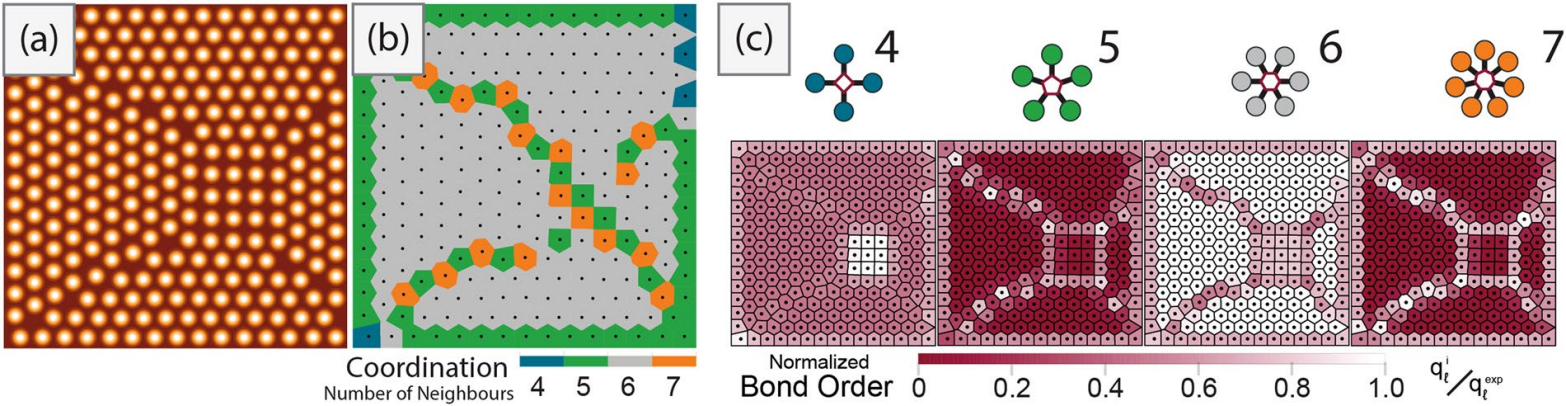
(**a**) Simulated micelles in a confined square boundary. (**b**) Voronoi tessellation of the micelle centroids coloured by the number of shared facets. An observer may expect particles in the center to have 4 neighbours, but this is not the case for the coordination number. (**c**) Voronoi tessellations coloured by the normalized bond order parameter for the type of symmetry (above). Whiter areas indicate particles that have high angular order in that symmetry basis. Particles with 5 and 7 neighbours highlight the grain boundaries and dislocation lines in their respective symmetry basis. The square configurations are not detected in the Voronoi tessellation as having 4 neighbours; however, they can be seen within the bond order with symmetry $\ell =4$.

For our micelle systems, which have roughly hexagonal symmetry arrangements, the coordination number for each particle is expected to be six (6) indicated in gray. All three systems show regions with the gray Voronoi cells of high hexagonal entropic order, within a larger area that is not globally perfectly hexagonal, (as already seen from the pair correlation analysis from section 3.1). With defects or local disorder, the steric frustration at the boundaries between large hexagonal sections will form pairs of Voronoi cells with alternating 5 and 7 sides. These are the so called “disclination” defects^58^, where the local number of neighbours is violated. In an analogy with dislocations, which is a defect in positional order, a collection of such defects can define “grain boundaries” or areas of higher local free volume, between entropically ordered sections of the spatial distribution^59^ (see Fig. 5(b)). Further frustration of the free volume minimization can result in even further deviations from the ideal coordination number, with 4 or even 8 neighbour particles suggesting large entropic disorder.

The coordination histograms from Fig. 4 indicates that the lowest spin speed (2000 rpm) results in relatively more six neighbour particles than at higher spin speeds, supporting the finding from the positional order that the system is basically hexatic. Using the coordination number frequency alone, however, suggests that the higher spin speeds are basically indistinguishable since the probability between having six neighbours and either 5 or 7 neighbours are similar.

The first neighbour maps (top right hand side) in Fig. 4 show how likely it is for the configuration to have particles with neighbours in a specific arc symmetry. This bond order^52^ or entropic valence^48^ diagram is a similar representation to a 2D pair correlation function^31^ and has been used to measure disordered magnetic moments^60^.

To build such a map, all particles are moved to a common origin, bringing with them the relative structure of neighbouring entropic “bonds” i.e. the coordination number and arc-separation angle for each particle. Each neighbour contributes a set of points to build up a probability distribution in space. The rigid objects that describe the entropic distributions are then rotated to have a common orientation. The position and angle of each neighbour is then plotted as a probability map, outlining the areas of preferred relative positions. The number of points each particle contributes is equal to its local coordination number. Regions of bright spots indicate higher chances of neighbours being at these positions.

Using the probability maps in Fig. 4 (top-right panel), it is now clear that the highest spin speeds lead to an isotropic distribution of particles, with the probability spread with little trends in angular order. On the other hand, the samples prepared with 2000 rpm and 6000 rpm spin-speeds are in a hexatic state as shown by the evenly spaced intensity of points around the particle at angles of 2*π*/6.

The highest density entropic order is found if each neighbour is spaced evenly at angles of $\theta =2\pi /\ell$ (also known as the angular order, described in more detail in section 3.3). In such cases, the coordination number, $\ell$, defines an expected arc-periodicity for neighbours, and can be used to determine the angular symmetry of the system.

This angular map can be further broken down by isolating subsets of particles based on their coordination number and grouping them together for analysis of the relative structure of set numbers of neighbours. This can allow for a high level comparison between the specific angular ordering that happens at the local particle level. This is particularly useful for the 5- and 7-fold disclination defects to indicate if there is some correlation between defects, through their angular distribution. Using this approach, therefore, exposes the hidden rotational symmetry of defects. These subset entropic valence maps are shown for the 5-, 6- and 7-neighbour angular distributions in the middle panels of Fig. 4. The common origin is coloured to identify the expected coordination number, using the colour scheme from the Voronoi tessellations.

For both the lowest and highest spin speeds, the five fold defects have no clear separation of intensity at the expected angles, suggesting that they are randomly distributed over the surface. However, at the intermediate spin speeds, some symmetry can be observed for the five fold defects. This suggests that the defects are clustered together, leaving large areas of entropic hexagonal order in the system.

Using this entropic order analysis based on the coordination number and entropic valence maps, the various structures can be classified as follows: the low spin speeds yield a mesophase structure of high hexatic order, with random defects. The intermediate spin speed results in a “polycrystalline” system, with defects segregated to “grain boundaries” between regions of higher order. The Voronoi tessellation diagram indicates that there is one large hexagonal region, surrounded on three sides by areas of higher free volume. Higher speeds ultimately prevent the formation of even those regions, with random defects distributed randomly over the surface.

By using the Voronoi tessellations and entropic valence maps, it is clear that lower speeds are more favourable to extracting a mesophase hexatic system, where entropic order is preserved even though translational order is lost, as we desired in our fictitious goal. By segregating the ensemble into portions using the coordination number, the amount and distribution of defects can be extracted to separate specific local information from the ensemble average.

### Detecting angular symmetry using the localized bond order

As discussed above, the highest density entropic order is found if each neighbour is spaced evenly at angles of $\theta =2\pi /\ell$, where $\ell$ is the entropically or enthalpically derived coordination number. Angular orientation order describes the likelihood of finding an object at a given angular arc-separation between neighbouring particles, most commonly thought of as the symmetry state of a system.

Symmetry can be understood in the context of periodical translational order, where objects are separated by fixed distances, in a given direction. The Bravais lattices are defined by type, number and direction of the allowable translations defining the symmetry space group (i.e. in a square lattice, objects can have a neighbour above, below, to the left or to the right, yielding four-fold symmetric allowable translations). Similar to the description of entropic order, the coordination number or number of nearest neighbours, $\ell$, drives the angular order, and this was taken advantage of in the previous section to describe the correlation of defects. Unlike for entropic order, which was related to the minimization of free space, the angular order relaxes the condition of maximal density; however, it imposes the condition that each neighbour is spaced evenly at angles of $\theta =2\pi /\ell$.

Humans are particularly adept at recognizing symmetry, as it is the basis of human pattern recognition^61,62^. If there are only a few particles or the positions have not deviated too far from the expected lattice positions, observers can usually extrapolate and distinguish if there is a different local symmetry at a given point (see Fig. 2(c and f). This perception and correct classification is highly dependent on a robust and consistent definition of the coordination number. However, as described in section 3.2, algorithms are sometimes limited in their ability to distinguish neighbours by interpolating localized periodicity in the same way an observer might. Simultaneously, observers may introduce bias by selecting or rejecting improper neighbours though inconsistency. Unwanted bias by either approach can be introduced using only bond-network neighbour descriptions, such as the Delaunay neighbour definition for the coordination number^56^. To overcome this problem, the bond order parameter ${q}_{\ell }$ can be utilized together with the coordination neighbours to explicitly differentiate particles which have the arc-symmetry we wish to tailor experiments toward. Specifically, we use the Voronoi-weighted Minkowski definition of the bond order parameter^56^, which reduces the influence from neighbours that have metastable edges.

Defining the bond order in this way provides a benefit for researchers who are exploring particle arrays that have ordering arc-symmetries other than that for $\ell \mathrm{=4}$ (i.e. square lattices) and $\ell \mathrm{=6}$ (i.e hexagonal lattice). Sections of particles with local 5-fold symmetry have been observed along disordered edges of self-assembled hard-spheres^63,64^. Also, planar arrays of colloidal particles subjected to quasiperiodic light fields of 5-fold arrangement of lasers have been shown to direct the self organization into quasicrystalline colloids^65,66^. These are dispersions of particles where the mix of multiple odd-integer symmetries of the lattices form complex structures that could easily be mis-classified by observers. In the next example, we emphasize a model system with two types of order to highlight the utility of using the bond order in **disLocate** to explore these hidden symmetries as $\ell$ is varied. This is done to precisely outline this complex behaviour, since in disordered material the effects are much more difficult to resolve.

In Fig. 5(a), we present a simulated configuration of (*N* = 256) micelle particles under square confinement^41^. This type of self-assembly causes internal frustration of the particles and leads to a different ordering in the center than that around the wall^67^. Hexagonal ordering can be observed at the walls while the square configuration imposed by steric frustration is in the center. As before, Voronoi tessellation of the centroids was taken and plotted using the colour scheme that highlights the coordination number (Fig. 5b). The majority of the system turns gray (6 neighbours) while disclination lines of alternating green/orange (5 and 7 neighbours respectively), separate the ordered “grains”, originating at the corners. The steric frustration from interacting neighbours at arc-angles different than the expected 2*π*/6 results in the local defects with 5 and 7-fold rotational symmetry. One thing that clearly stands out is the lack of Voronoi cells with 4 sides (dark blue) at the center. One consequence of using the coordination number definition for neighbours is that it may incorrectly over-count due to the metastable square Voronoi cell^68^. In this case, subjective classification of local symmetry due to steric frustration is quickly seen while the numerical definition inaccurately identifies false neighbours using the expected particle spacings.

Figure 5(c) shows an overlay of the bond order parameter in symmetry basis $\ell =4,5,6,7$. These values are normalized by the expectation value for the given symmetry (see last entry of supporting information Table SIII) onto the same Voronoi tessellation shown in the coordination map in Fig. 5(b). Here, the dark shade represents bond order parameters that are heavily deviated away from the expected value while lighter and whiter sections are ones where they are closer to expectation values in that symmetry basis. Separating the rotational symmetries allows for an observer to judge the reliability of the internal structure as described by metrics using the coordination definition of neighbours. The expectation values for the hexagonal and square lattices are given in Table SIII (Supporting information).

In general, 6-fold bond order values (*q*_6_) are reported in research without consideration for other symmetry values, as close packing of circles yields a hexagonal configuration. Looking only at *q*_6_ in Fig. 5, most of the system is closer to white, suggesting that the system is relatively hexagonal as expected. The mean bond order parameter value in Table SIII (Supporting information) also gives a similar indication – that the system is mostly hexagonally ordered, with some disorder. One consequence with using only *q*_6_, however, is that disordered sections can take on values similar to that of the square pattern in the center, making it difficult to classify if there is angular ordering other than the 6-fold – all are just deviations from the expected value of *q*_6_ for a hexagonal lattice. Table SIII (Supporting information) shows how different symmetry operations can take on non-zero values even for perfect lattices. Using the Voronoi tesselation coloured by 4-fold bond order values, *q*_4_, the 4-fold square configuration in the center can be isolated, as shown in the left-most panel of Fig. 5c.

Disordered systems have variations in all their intermolecular structures. There will be high probability for particles with coordination other than six, dispersed throughout the sample. Disordered states should produce bond order values that converge towards a similar value in all symmetry basis. Table SIII (Supporting information) lists the values for a simulated dispersion of (*N* = 5000) pseudo-random point-particles to show the possible values when examining the fully disordered system. When dealing with disordered states with partial internal ordering, however, there can be correlations or distributions that are not captured solely by the single mean value (see Fig. 4). Experimentally, 5-fold localized structures have been observed to have correlations in partially disordered systems where 6-fold symmetry is expected^69^. The exploration of multiple different symmetry types can reveal information often missed by either observers and/or computers^70^ (also see section 3.2). In Fig. 5c, the 5-fold pentagonal configurations, *q*_5_ matches almost exactly to the calculated 5 sided Voronoi cells. The 7-fold bond order parameter similarly targets the cells with coordination number of 7. The combination of 5 and 7-fold bond orders highlight the disordered boundaries that separate the square and hexagonal crystallites from each other, targeting the transition between highly ordered hexagonal and square states.

With that general framework, we can apply the same approach to the experimental micelle spin-speed series data. Supporting information Table SIII reports the mean values for the normalized bond orders for the three experimental data sets. For any given data set, the values do not seem very different from the randomly generated pattern. However, the trend in changing bond order parameters as the spin speed increases does give some insights into the angular ordering behaviour.

Using only *q*_4_ and *q*_6_, as would be a common approach in the literature, suggests that low spin-speed produces relatively hexagonal packings with some low probability for square packing. By increasing this speed, the limited data set would suggest that the system transitions from hexagonal into a square lattice (as determined by the decrease in *q*_6_ and increase in *q*_4_). However, it is clear that *q*_5_ and *q*_7_ also increase with increasing spin speed. In general, if there is an increase in order or the transition from one type of order to another, higher ordered states will converge to the expected value for the maximum symmetry while decreasing all others. On the other hand, the bond order values converge towards a similar value in all symmetry bases for disordered states, due to the broken local symmetry. This would also appear as an isotropic distribution of intensity in the entropic force map (see top panels of Fig. 4) for disordered systems; for ordered systems, the new symmetry state would emerge in the probability map (i.e. a square pattern would emerge if there were a transition from hexagonal to square). Therefore, from the trend of all four parameters, it can be concluded that increasing speeds cause a loss of hexagonal order in favour of more localized disorder, supporting the conclusions using the previous tools.

For the experimental data, the Voronoi maps coloured with the localized bond order (see supporting information Fig. S7) reflect the trends already identified – lowest spin speeds result in the largest regions of hexagonal order; as spin speed increases the disorder becomes randomized. The *q*_7_ map, which is more useful in highlighting grain boundaries, gives a little more insight compared to the entropic analysis alone – the random defects at the lowest spin speed are actually more like a “grain boundary’ separating two large hexagonal regions.

By using the Voronoi tessellations and the bond order parameter, it is again clear that lower speeds are more favourable to extracting a mesophase hexatic system, where angular symmetry is preserved, as we desired in our fictitious goal. By examining the ensemble with both global and local bond order parameters of various arc-symmetry, the nature of the defects at lower speeds can be determined to be analogous to “grain boundaries” separating regions of higher order; at high speeds, due to the random distribution of defects, these dispersions are closer to complete spatial randomness.

### Concluding remarks on combining numerical methods and visualizations

Structural characterization is a key aspect of reproducible experiments which allows for critical insight into the external factors which drive self-assembly. Robust comparisons between similar samples, especially with limited data sets, need a finely tuned ensemble of accurate analysis tools. We have combined a range of tools as a freely distributable Mathematica package (disLocate.m) which can be applied to any data set that contains spatial information.

Using the full range of tools available in **disLocate**, we examined as a representative example of AFM images of polystyrene-block-poly-2-vinylpyridine (PS-b-P2VP) diblock copolymer micelles distributed on a Si wafer surface with three different deposition approaches. From an analysis of the structures, we are able to distinguish them with a high degree of specificity, from a variety of perspectives. To illustrate the power of the tools in allowing a user to come to critical decisions about experimental protocols based on limited information, we established a fictitious goal: can the spin speed alone be used to increase the internal spacing of two micelle diameters between particles, while maintaining mostly hexagonal angular order, separated by limited regions of disorder or defects, making the surface polycrystalline. Based on our analysis of all the global mean and local variance information on the structure of each data set, it is clear that further experiments with varying spin speeds would not lead to our desired outcome, due to the conflicting influences on the various types of order in the micellar system. The slower spin speeds encourage the formation of a nearly hexagonal order, with some contained defects, but brings the particles closer together to increase the entropic order. Higher spin speeds are able to increase the spacing, but at the expense of angular order, resulting in a system that appears almost like complete spatial randomness. An intermediate spin speed seems to fulfill most of the requirements set out. Even though there are similar numbers of defects as at higher speeds, there are still regions of high hexatic order. However, this number of defects may be too high, as the hexagonal regions are about the same size as the disordered regions. Each piece of information gained by the tools gave different insight into the type and quality of the order. Based on the analysis provided by **disLocate**, we were able to narrow our the choices for next methodological improvement depending on the desired importance of each ordering. If hexagonal dispersions are highly desired, then slower spin speeds are needed. If the priority is for larger intermolecular spacing, then higher speeds are necessary. To achieve different types of ordering with a density that was observed, then other factors may need to accompany the refinement in spin speeds (like temperature, or solvents) when it is set between these two ranges. By identifying that increasing speed will increase the intermolecular spacing at the expense of the angular periodicity, using **disLocate** for spatial analysis allows us to quickly change experimental tactics by providing an accurate trend with a limited amount of data.

Though we used AFM micrographs as the basis for extracting the spatial organization, our package relies solely on the x-y coordinates of the centroids of the particles. As such a point pattern can be derived from any image, **disLocate** provides a valuable tool for quantitative microscopy across any length scale. Using the tools in the **disLocate** package, different structure metrics of single image patterns can be compared to quantify local variation in disorder through a variety of methods. Though researchers may use one or some of the tools outlined in this contribution, the complete package allows one to easily and rapidly extract all of the different parameters in Table 1 to make robust conclusions on the order of a given system. Each state variable can be defined either as a single global description or as a local average value with uncertainties determined using internal variation as suits the intended purpose. We strove to remove unintentional user-introduced bias by advocating and implementing metrics based on the local variation in Voronoi tessellations as a way to promote consistency within the numerical analysis and interpretation of state variables between experimental setups. Using this package, which is freely available online, researchers can quickly and easily quantify the experimental trends which produce desired morphologies and engineer novel methods which can direct self-assembly.

## Methods

### Software Availability

The code included with this manuscript is written as a (*.m) Mathematica Package. It currently supports Version 10 and above. Some highly optimized functions may require a local C compiler (such as Visual Studio, Clang, etc) for execution at full speed. Calling the package may cause a warning to appear, but these functions will still run without the compiler. The package uses as inputs x-y coordinates for the centroids of any point pattern of objects. The data formats that are compatible with our package are.csv (comma separated values),.xls (excel spreadsheet files), and.dat (arbitrary tabular data). The user manual is included with the package.

### Data Availability

The raw AFM data that was used in this manuscript is bundled as example data inside the dislocate.zip file in Supporting Information. It also includes the xy positions extracted from imageJ and the height profiles from post-processing.

### Experimental details

Polystyrene-block-poly-2-vinylpyridine (PS-b-P2VP) diblock co- polymer (Polymer source, P1330-S2VP) was dissolved in o-xylene (CALEDON) to form core-corona reverse micelles and kept under vigorous stirring for 18 h. Dynamic spin-coating (Speciality Coating Systems, SCS G3) at 2000, 6000 and 8000 rpm was used to produce a 2D monolayer array on 1 cm Si(100) substrates with native oxide, diced from 6” wafers. The substrates were cleaned with acetone and ethanol in an ultrasonic bath, rinsed with deionized water, and dried in an *N*_2_ stream. Images of the micelle dispersions were analyzed using atomic force microscopy (AFM) in air in tapping mode with a phase locked loop (PLL) dynamic measurement board (Asylum). The non-contact silicon tips were OTESPA (Asylum) with a resonance frequency of 300 kHz, a force constant of 26 N/m and a tip radius of curvature <7 nm. The AFM images were processed with WSxM (NanoTec).

### Pair Correlation Function

The pair correlation function is a tool to analyze positional order. It is calculated using the probability of all distances between particles internal to the system. Every particle has a list of distances from its centre to its sequential nearest neighbouring shells. An initial central particle *a* is chosen as the origin. A circular shell of width *dr* expands from the center to a radius distance *r*. Any particles inside a circular shell of width Δ*r* are counted together and binned to produce the neighbour probability *n*_*n*_(*r*) as a function of distance, weighted by the particle density, *ρ*.

1 $$g(r)=\frac{{n}_{n}(r)}{2\pi r{\rm{\Delta }}r\rho }$$

As such, this function is sometimes described as the “radial distribution function” since it counts the distribution of neighbours over the radial spatial dimension. With this definition, information on angular orientation is lost in favour of positional probability.

The pair correlation function is not very well defined when samples consist of less than a few hundred particles. The micelle samples analyzed here are these kind of systems. To overcome these challenges, we have implemented a bootstrap technique to generate similar artificial systems with slight deviations. Each micelle configuration was subjected to the same procedure used to define the hexatic lattice, where the particles are randomly displaced around their centres by a fixed standard deviation. This value is associated with the experimental error, where particle centres are uncertain to within one pixel. Ensembles can be generated from randomly displacing the particles by the experimental error. For the micelle case, this ensemble was generated with 1000 independently shaken patterns with a Gaussian probability distribution using variance of half the experimental pixel size width (2.44 nm/pixel). This technique produces data that is analogous to multiple observations of the same area over different time periods. This allows for the smoothing of sharp features in the *g*(*r*) which are artifacts of the finite number of intermolecular distances. Slight variations in each observation will produce a set of particle coordinates that are not exactly identical. This is a key aspect in removing spurious peaks in the pair correlation function caused by finite data sets. These ensembles are solved together in a global average *g*(*r*), the small displacements allow particles to hop between bins which smooths the probability distribution.

### Hexatic Lattice

Density is correlated to the average intermolecular spacing between the centroids of particles. Generating a proper periodic point pattern for an accurate comparison reference depends on a few key components. One is the number of particles (*N*) inside the window and the other is the area encompassed by that window (*A*_*box*_). This number of particles per area defines the intensity, *λ* = (*N*)/(*A*_*box*_), and can be used to calculate the average spacing between neighbouring particles in the hexagonal state.

The goal is to generate a hexagonal or close to hexagonal pattern with the same intensity as the target pattern as a basis for a comparison of the order of the target pattern. The total area of all the Voronoi cells must equal the total area from which the number of particles has been observed $\frac{1}{{{\rm{\sum }}}^{{\rm{N}}}\,{A}_{{vor}}}$.

As the particles are represented purely by their centroids, the distance between two objects of any size and shape can be represented as close-packed (touching) circles with diameter (2*r*_*hex*_) which can be solved geometrically for the *hexagonal lattice spacing*. This equation is derived by solving for the apothem of a hexagon with area equal to the local Voronoi cell $({2r}_{hex}=({2/(\lambda {(3)}^{1/2}))}^{1/2})$. Each cell is defined, therefore, by a circular particle of radius *r*_*hex*_ that would produce a Voronoi cell with exactly similar area to the one solved at the particle location. In other words, the local hexagonal radius at location *i* is a function of local Voronoi area (*A*_*vor*_) only:

2 $${r}_{hex}^{i}=\sqrt{{A}_{vor}^{i}}{\mathrm{(2}\sqrt{3})}^{-\frac{1}{2}}$$

The resulting variation in local Voronoi volumes translates into a distribution of possible *r*_*hex*_ sizes, which can be estimated with a mean and variance. This mean is the effective distance where intermolecular interactions and density are equalized. The variation in this effective distance represents the relative positional agitation of each particle at their location (i.e. the entropic portion of random local movements). The uncertainty in mean intermolecular distance (Δ*r*) can be calculated using a method of error propagation, using the Voronoi cell areas as the independent variable in the lattice spacing function (*r*_*hex*_) and the standard deviation (Δ*u*_*r*_).

3 $${\rm{\Delta }}r=\sqrt{{(\frac{\partial ({r}_{hex})}{\partial {A}_{vor}}{\rm{\Delta }}{u}_{r})}^{2}}$$

The result can be modeled as a system where particles have a global mean spacing (2*r*_*hex*_) but also fluctuate around the center (*r*_0_ = 0) with a mean square displacement proportional to the local density variation.

$${({\rm{\Delta }}r)}^{2}={\langle r-{r}_{0}\rangle }^{2}=Var({r}_{hex})$$

### Bond Order Parameter

The bond order parameter uses the angles between neighbours to calculate a metric that describes their arc distribution relative to a central particle. A reference frame needs to be chosen to properly assign angles and typically the horizontal (x)-axis is a common choice. Rotational symmetry can be exploited to define the bond order parameter in terms of any arbitrary axis using spherical harmonic functions. Consider a particle *a* which has a set of nearest neighbours *n*_*n*_ = (*b*, *c*, *d*, …) containing *a*_n_ neighbours . Each neighbour is at an angle ${\theta }_{{a}_{({n}_{n})}}$ relative to the reference frame vector and the neighbour vector.

4 $${q}_{\ell }(i)={[\frac{4\pi }{2l+1}\sum _{m=-\ell }^{\ell }{|{Y}_{\ell m}|}^{2}]}^{\mathrm{1/2}},$$

where ${Y}_{\ell m}$ is spherical harmonic function with degree $\ell$ and order *m*.

5 $${|{Y}_{\ell m}|}^{2}=\,{|\sum ^{{n}_{n}}\frac{1}{{n}_{n}}{Y}_{lm}(\theta ,\phi )|}^{2}$$

In the case of neighbours being defined using Voronoi coordination, the value of *n*_*n*_ is replaced by a weighting function, determined by the length of the Voronoi facet (*p*_*a*_) passing through its bond vector connecting the central particle to its neighbour. This is then divided by the total sum of Voronoi facets from neighbours^56^.

6 $$\frac{1}{{a}_{n}}=\frac{{p}_{{a}_{b}}}{\sum _{i}^{(b,c,d,\ldots )}{p}_{{a}_{i}}}$$

This indicates that the weighted probabilities remains at unity when summed over all neighbour particles, The configuration average is the mean value of each local bond order associated with the particles in the system.

7 $$\langle {q}_{\ell }\rangle =\frac{1}{N}\sum _{i}^{N}{q}_{\ell }(i)$$

The normalized bond order is solved by dividing the mean bond order by the bond order associated with the highest neighbour configuration symmetry ${q}_{\ell }^{{\rm{sym}}}$ which has $\ell$ neighbours with arc separation angle $(\theta =2\pi /\ell )$.

## Electronic supplementary material

Supporting Information

Supporting Information 2
