# Author Correction: disLocate: tools to rapidly quantify local intermolecular structure to assess two-dimensional order in self-assembled systems

**DOI:** 10.1038/s41598-018-24171-y

**Published:** 2018-04-16

**Authors:** Matt Bumstead, Kunyu Liang, Gregory Hanta, Lok Shu Hui, Ayse Turak

**Affiliations:** McMaster University, Department of Engineering Physics, Hamilton, L8S 4L7 Canada

Correction to: *Scientific Reports* 10.1038/s41598-017-18894-7, published online 24 January 2018

An additional Supplementary Information file containing programme codes was omitted from the original version of this Article. This has been corrected and the additional Supplementary Information accompanies this Article.

