# Supplementary material for: disLocate: tools to rapidly quantify local intermolecular structure to assess two-dimensional order in self-assembled systems
Source: Sci Rep. 2018 Jan 24;8:1554. doi: 10.1038/s41598-017-18894-7 (PMC5784143; doi:10.1038/s41598-017-18894-7)

disLocate: tools to rapidly quantify local intermolecular structure to assess two-dimensional order in self-assembled systems

Matt Bumstead,[1] Kunyu Liang,[1] Gregory Hanta,[1] Lok Shu Hui,[1] and Ayse Turak[1,*]

[1]*Department of Engineering Physics, McMaster University, Ontario, Canada*[†]

(Dated: November 14, 2017)

I. SUPPORTING INFORMATION

This supporting information document contains a number of tables for structure and order parameters discussed in the main text for the experimental data of the micelles with various spin speeds. It also includes three examples of microscopy data at varying length scales that can be analyzed using the **disLocate** package.

A. Example microscopy data

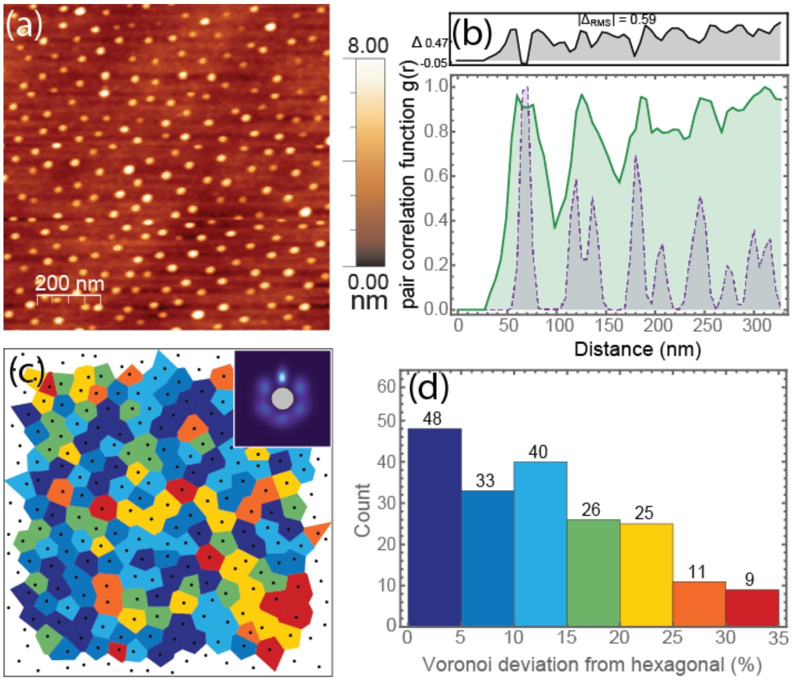

FIG. S1: (Color online) (a) AFM micrographs of FeOx nanoparticles (b) Pair correlation function showing hexatic (six-fold) structure of nanoparticle dispersion (c) Voronoi tessellation of nanoparticle positions. Each cell colored by percent deviation off the expected cell produced from hexagonal packing. Inset shows the orientational order map for all particles with six neighbours, similar to an FFT showing the periodicity (d) Histogram of Voronoi deviations from (c).

*Corresponding author: turaka@mcmaster.ca
†Corresponding author: bumstema@mcmaster.ca

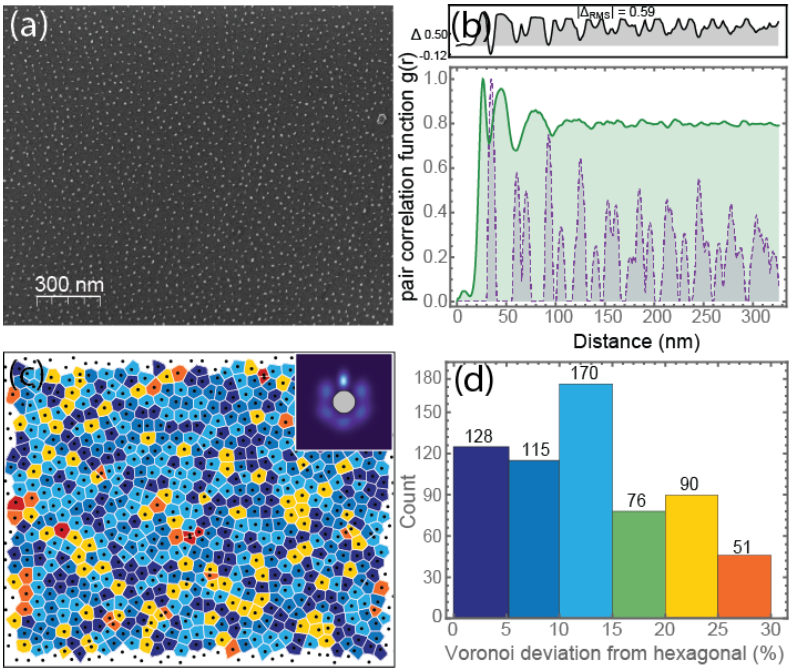

FIG. S2: (Color online) (a) SEM micrographs of LiF nanoparticles (b) Pair correlation function showing hexatic (six-fold) structure of nanoparticle dispersion (c) Voronoi tessellation of nanoparticle positions. Each cell is colored by percent deviation off the expected cell produced from hexagonal packing. Inset shows the orientational order map for all particles with six neighbours, similar to an FFT showing the periodicity (d) Histogram of Voronoi deviations from (c).

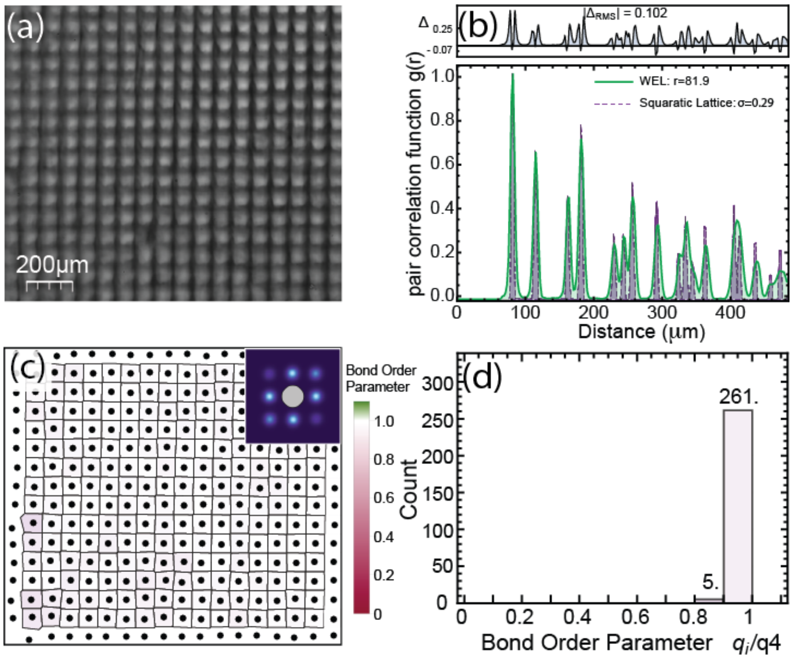

FIG. S3: (Color online) (a) Optical micrograph of waveguide encoded lattice arrays (WEL) formed by the self-trapping of light filaments in a photoactive polymer, supplied by K. Saravanamuttu (ref. Hosein, Adv. Funct. Mater. **27**, 1702242 (2017)). (b) Pair correlation function showing square (four-fold) structure of the filament dispersions (c) Voronoi tessellation of the waveguide positions. Each cell is colored by the bond order normalized to the expected value for q_4, the square lattice. Inset shows the orientational order map for all nearest neighbours, similar to an FFT showing the periodicity (d) Histogram of Voronoi deviations from (c).

B. Data extracted from the AFM micrographs for various spin coating speeds of diblock copolymer micelles on Si

TABLE SI: Comparing various global methods which calculate the number density. N/A is the number of particles per micron squared (μn^2) which is the area of the image. r is the mean radius in nanometers (nm). ϕ is the covering area. The number N_{vor} of particles decreases by removing edge artifacts. The Voronoi areas are used to determine the expected number per micron N_{vor}/A_{vor}. The standard deviation in Voronoi area is used to estimate the number variation $\sigma(N_{vor}/A_{vor})$.

Micelle Data	N/A	$2r$	ϕ	N_{vor}	N_{vor}/A_{vor}	$\sigma(N_{vor}/A_{vor})$
2000 rpm	183	52	0.32	132	169.2	12.8
6000 rpm	126	58	0.27	88	114.9	11.7
8000 rpm	107	53	0.21	71	99.6	12.6

TABLE SII: Comparing various methods which calculate the first-neighbour intermolecular spacing. This distance is determined using the first peak of the pair correlation function $g(r)^{1^{st}}_{\max}$, the expected spacing from hexagonal lattice $2r_{hex}$ with the same global density as the micelle images, the mean expected spacing $\langle 2r^{vor}_{hex}\rangle$ from the Voronoi density located at particle positions, and the internal variance of spacing Δr which was calculated using the variance of local Voronoi areas. Distances are reported in nanometers (nm).

Micelle Data	$g(r)^{1^{st}}_{\max}$	$2r_{hex}$	$\langle 2r^{vor}_{hex}\rangle$	Δr
2000 rpm	79.8	79.4	82.7	2.5
6000 rpm	91.7	95.7	100.2	6.4
8000 rpm	100.5	103.8	107.6	11.4

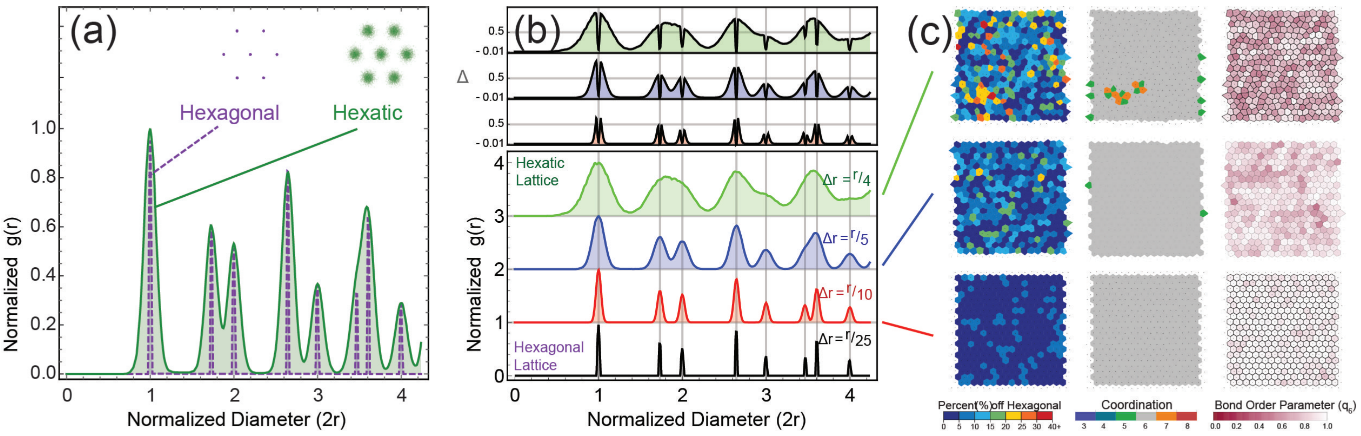

FIG. S4: (Color online) (a) Normalized pair correlation function for a perfectly hexagonal lattice and for a hexatic lattice defined by lattice disorder parameter Δr of $r/5$ deviations to the lattice positions. (b) Normalized pair correlation functions for hexatic lattices defined by various lattice disorder parameters, offset for clarity. The inset shows the difference spectrum between the hexatic lattices and the hexagonal lattice defined by delta functions at the bottom. (c) The corresponding Voronoi tesselations for the hexatic lattices colored by the percent deviation off the expected cell produced from hexagonal packing with the same density, the coordination number, and the bond order normalized to the expected value for q_6, the hexagonal lattice.

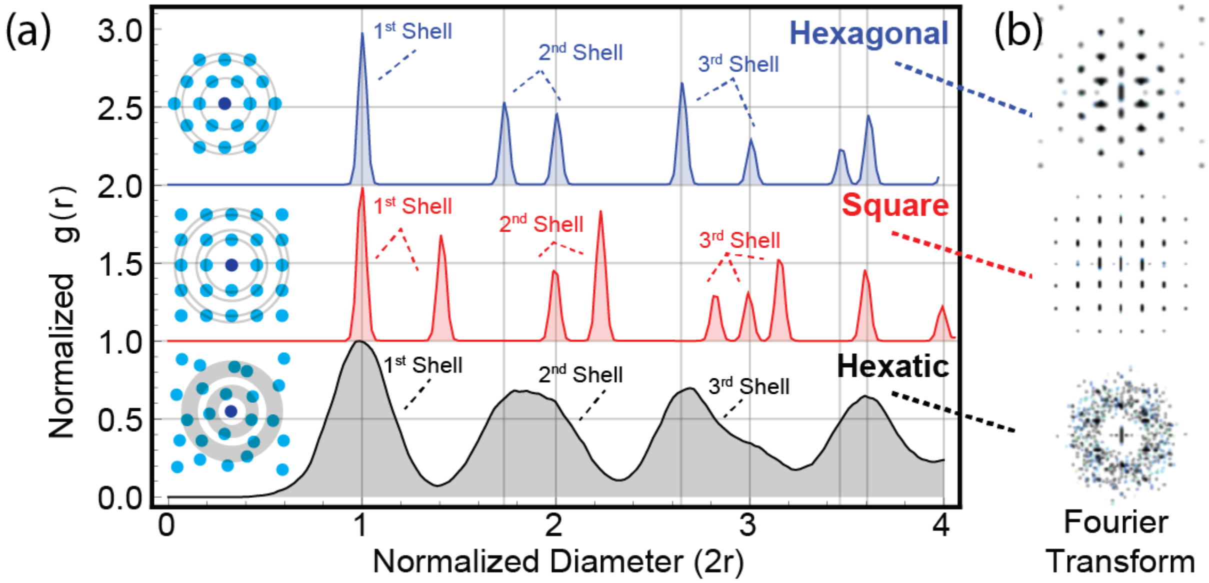

FIG. S5: (Color online) (a) Normalized pair correlation function for hexagonal, square, and hexatic lattices (b) Fast Fourier Transform (FFT) of the point patterns corresponding to each distribution of points.

TABLE SIII: Mean bond order with angular symmetry ℓ, $\langle q_\ell \rangle$ for disordered micelle systems. Non-zero values for q_ℓ appear within configurations even though they have zero particles with the same coordination number ($\ell = 4$).

Pattern	$\langle q_4 \rangle \ / q_4^{\mathrm{sq}}$	$\langle q_5 \rangle \ / q_5^{\mathrm{pent}}$	$\langle q_6 \rangle / q_6^{\mathrm{hex}}$	$\langle q_7 \rangle / q_7^{\mathrm{sept}}$
Hexagonal Lattice	0.4523	0	1	0
Square Lattice	1	0	0.7914	0
Simulated - Fig 5c	0.5310	0.2572	0.8602	0.3182
Spin 2000 (rpm)	0.5434	0.4169	0.7705	0.5261
Spin 6000 (rpm)	0.5329	0.5087	0.6896	0.6231
Spin 8000 (rpm)	0.5868	0.4952	0.6887	0.6253
Random	0.6504	0.5615	0.7055	0.6438
Normalize: $q_\ell^{\mathrm{sym}} =$	0.8290	0.7016	0.7408	0.6473

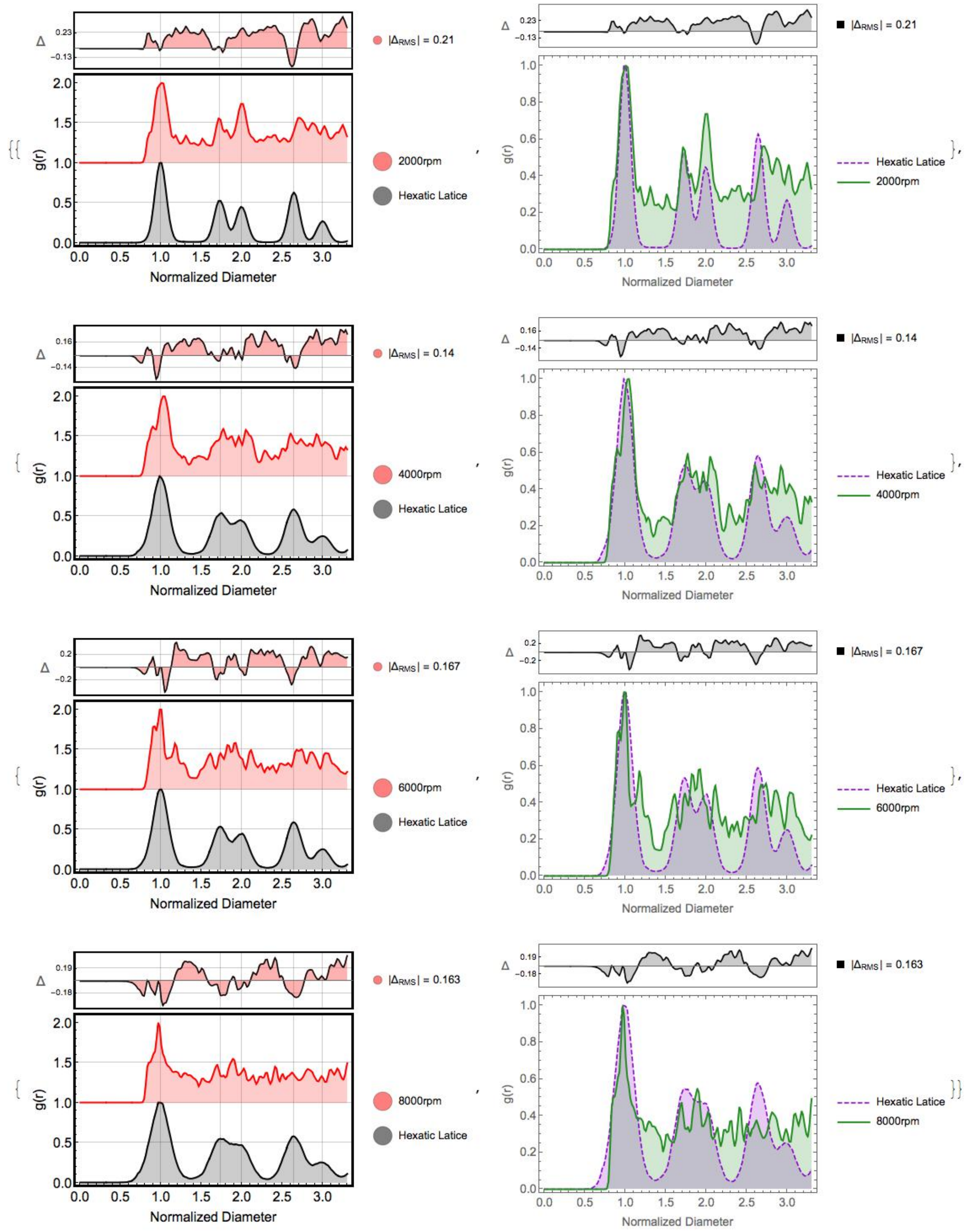

FIG. S6: (Color online) Normalized pair correlation function comparisons extracted from the AFM micrographs of micelles with various spin speeds with the equivalent hexatic lattice determined from the lattice disorder parameter. Left hand side shows a stacked representation of the comparison, whereas the right hand side shows an overlay of the same data.

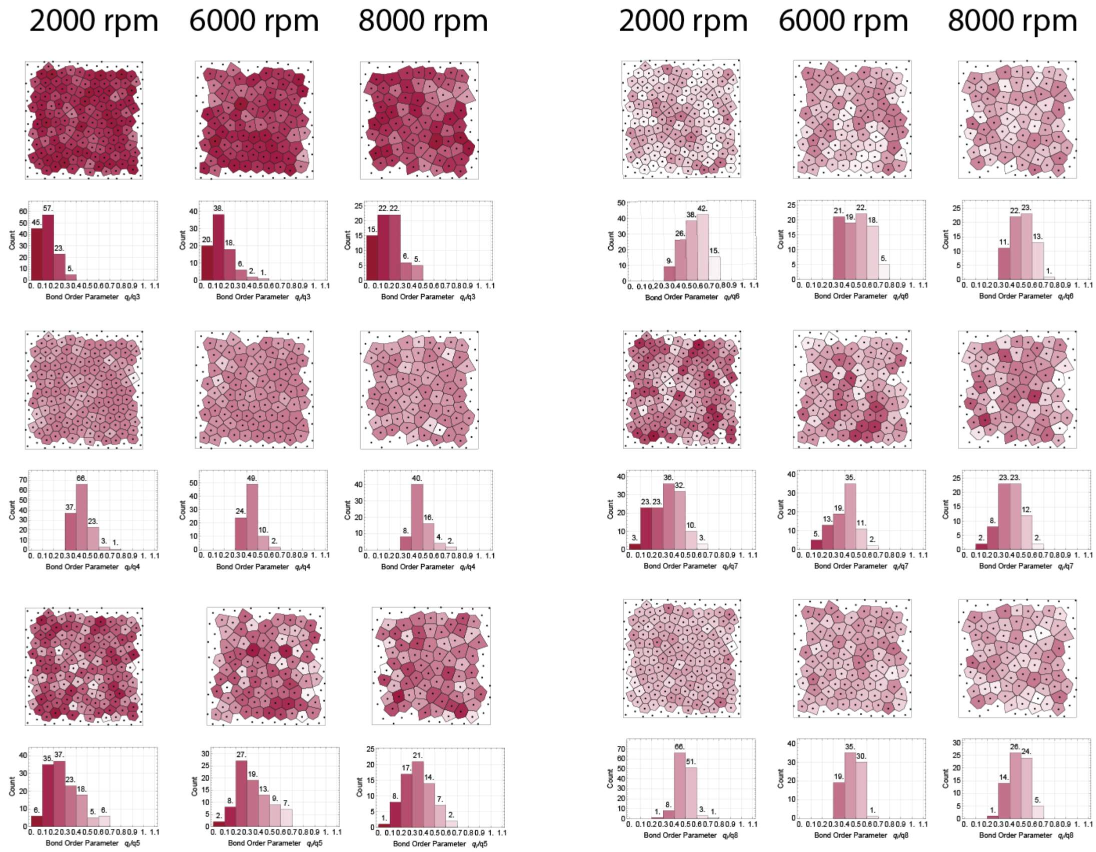

FIG. S7: (Color online) Voronoi tessellations coloured by the normalized bond order parameter for the type of symmetry. Left hand side q_3, q_4, q_5. Right hand side q_6, q_7, q_8. Whiter areas indicate particles that have high angular order in that symmetry basis.

disLocate - *Mathematica* Package:

(d)etecting (i)ntermolecular (s)tructure (Locate)d at particle positions

Load the disLocate.m Package into *Mathematica:*

1. Install Package:
Menu Bar > File > Install... > Type [Package] Source: [From File...] Name: [disLocate] > [OK]

Click the Blue Cell and Press the Two Buttons: “Shift” + “Enter”

In[122]:=
```
<< disLocate`
disLocateVersion
```

Out[123]= `1.2017-08-31.thesis-M.v11-final`

Note: Make sure this line produces a version number before continuing

Select and Import the Data Files

2. Run all Examples by clicking:
Menu Bar > Evaluation > Evaluate Entire Notebook

NOTE: Make sure this notebook is in the same directory as the data folder: micelle_data/

In[126]:=
```
dir = NotebookDirectory[] <> "micelle_data/";
SetDirectory[dir];
```

In[128]:= **images** = {
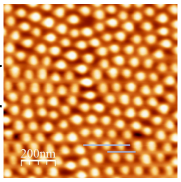

,
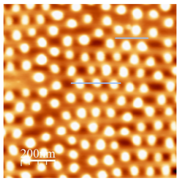

,
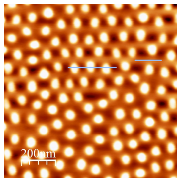

,
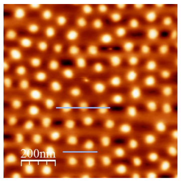

};

In[129]:=
```
xylene2000 =
  Import[dir <> "2000rpm/Result 2000rpm 1um.csv", "CSV"][[2 ;;, 3 ;; 4]];
xylene4000 = Import[dir <> "4000rpm/Result 4000rpm 1um.csv", "CSV"][[
   2 ;;, 3 ;; 4]];
xylene6000 = Import[dir <> "6000rpm/Result 6000rpm 1um.csv", "CSV"][[
   2 ;;, 3 ;; 4]];
xylene8000 = Import[dir <> "8000rpm/Result 8000rpm 1um.csv", "CSV"][[2 ;;, 3 ;; 4]];
```

In[133]:=
```
xyXyleneList = {xylene2000, xylene4000, xylene6000, xylene8000};
nameList = {"2000rpm", "4000rpm", "6000rpm", "8000rpm"};
```

Voronoi Tessellations - Area Distributions

```
? PlotVoronoiTessellations
```

PlotVoronoiTessellations[data2Din_, OptionsPattern[]]:= Returns a Graphics of the VoronoiCells[] that has been colour coded to associate disorder metrics to the planar arrangements.
OptionsPattern=(type→ToString[vor],symmetry→6,cells→{},points→True,scalebar→False,stats→ToString[Count],boundary→ToString[NOEDGE],box→{})

type–>ToString[voronoi]=The Main variable that changes the information provided. Option: type→ToString[voronoi] Uses the areas of the Voronoi Cells to calculate an expected hexagonal spacing with the same intensity (shortname ToString[vor]). Option: type→ToString[coordination] Uses the number of facets each individual Voronoi polygon has (shortname ToString[coord]). Option: type→ToString[BondOrder] Uses the neighbours –as defined by the Delaunaly Triangulation– to calculate the BondOrderParameter[]. Default symmetry is (symmetry–>6) but can be manually set to any variable.

symmetry –> 6 : Sets the symmetry number associated with the BondOrderParameter[]. No effect with any (type).

cells –> {} : This option provides a way to manually pass VoronoiCells[] that have been precalculated. It is meant to save time by passing the automatic VoronoiCells[] call for a large systems that might be run many times through this function.

points –> True : A graphics option that includes the centroids of the particles on the final plot. These can be turned off with this option.

scalebar –> False : An option to include the scalebar assocciated with the colour code of (type).

stats –> ToString[Count] : Sets the type of stats passed into VoronoiHistogram[]. Recommended settings – ToString[Count] or ToString[Probability]. See 'hspec' Option for Histogram[].

boundary –> ToString[NoEdge] : Settings for boundary corrections. Default boundary→ToString[NoEdge] automatically removes any Voronoi Cells that lay on or outside of (box). Options include: boundary→ToString[periodic] includes edge Voronoi polygons by adding periodically translated neighbours outside of the box (shortname pbc). boundary→ToString[image] includes edge Voronoi polygons by adding virtual particles that produce Voronoi cells with facets exactly on the edge of (box). This effectively truncates or clips the edges of the Voronoi cells to the (box). (shortname img).

box –> {} : Explicitly defines the edges of a box. Note! – setting a polygon will not change the (boundary), as it will default boundary→ToString[NoEdge] which autodetects the box from the data. Remember to set a (boundary) option.

```
(*  Basic Plot for the Voronoi Tessellation                         *)
(*  Returns the Image of the Tiles and the Histogram of the data *)

PlotVoronoiTessellations@xylene2000
```

{ 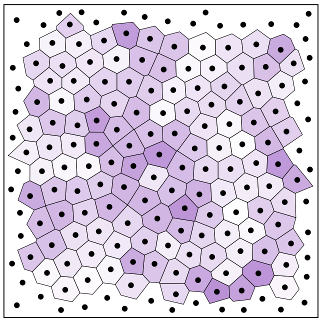, 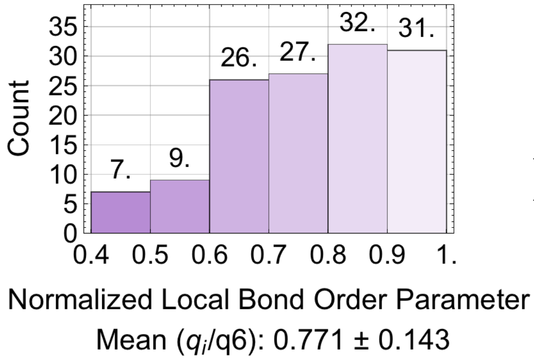

}

```
(* Additional Option #1 - type *)
(* Change the colour scheme of the Voronoi Tiles *)

PlotVoronoiTessellations[xylene2000, scalebar → True, raster → True, type → #] & /@
 {"vor", "bop", "coord"}
```

{{ 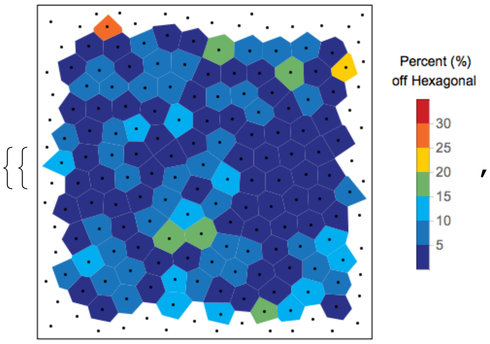

, 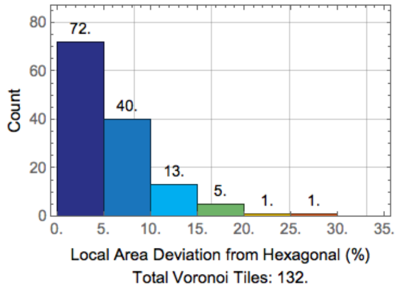

},

{ 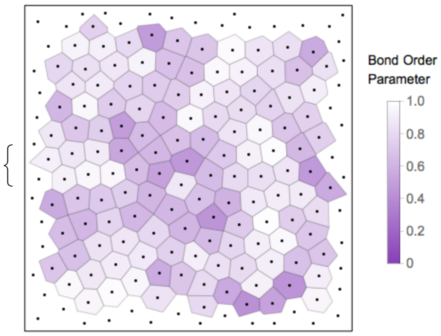

, 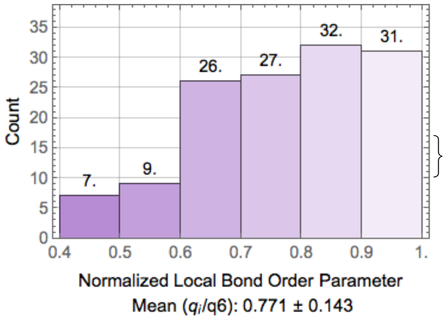

},

{ 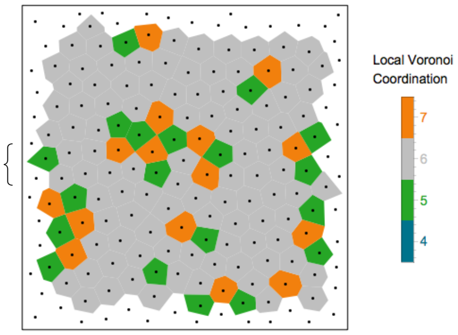

, 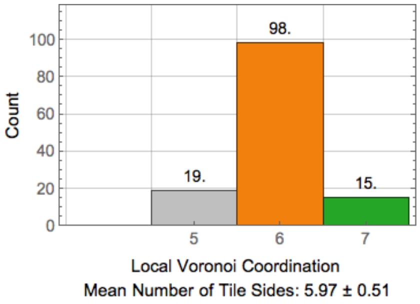

}}

```
(* Additional Option #2 - stats *)
(* Instead of Counting the Number of tiles, the Probability can be used. *)

PlotVoronoiTessellations[xylene2000, type → "vor", stats → "Probability"]
```

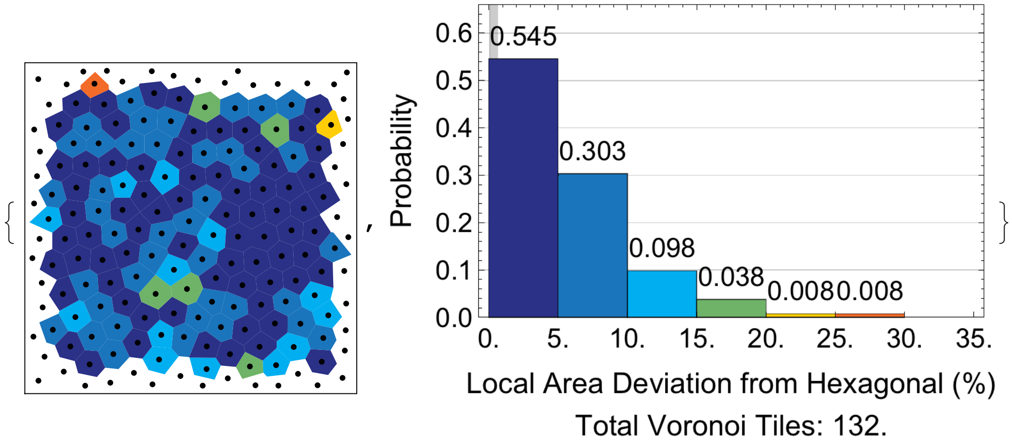


```
(* Additional Option #3 - points *)
(* Remove the points and lines to give it a more "stained glass" look *)

PlotVoronoiTessellations[xylene2000, type → "vor", points → False]
```

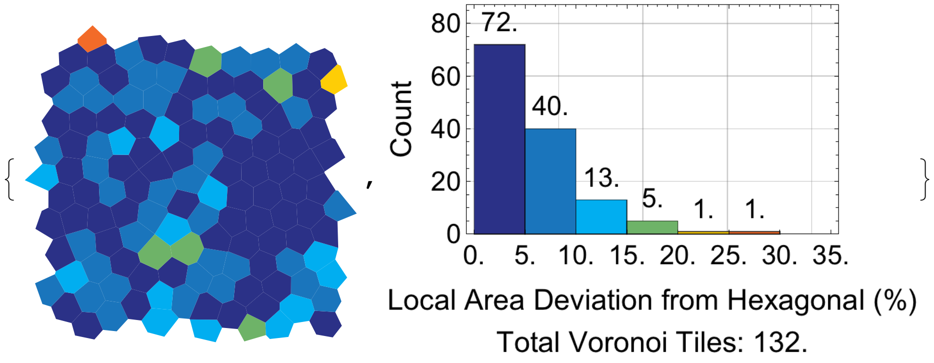

```
(* Additional Option #5 - boundary *)
(* Change the routine to sample points outside the edge *)
(* - noedge: removes any tiles that extend/touch the box *)
(* - pbc:  Applies Periodic boundaries to the edge,
total tile areas will total the box area *)
(* - image:  Places image charge particles
   outside so that the Voronoi tiles clip to the box *)

(* WARNING: type→"vor" depends on the global
  number density which changes with more particles *)

PlotVoronoiTessellations[xylene2000, type → "vor", scalebar → True,
   raster → True, boundary → #] &/@{"noedge", "pbc", "image"}
```

{{

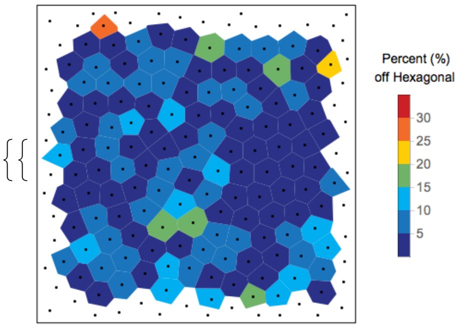

,

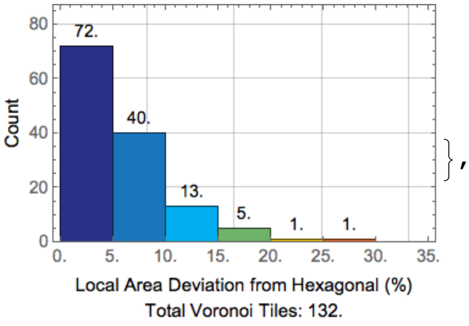

},

{

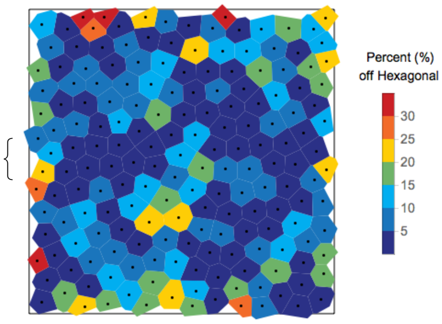

,

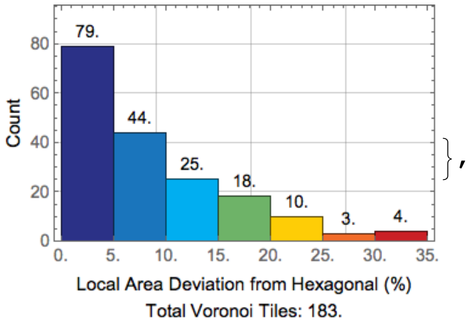

},

{

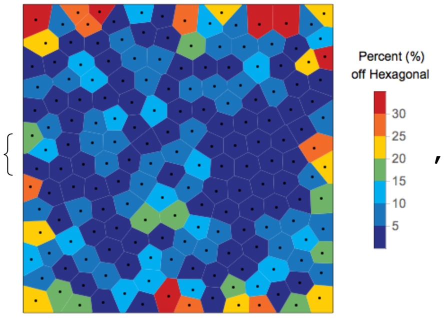

,

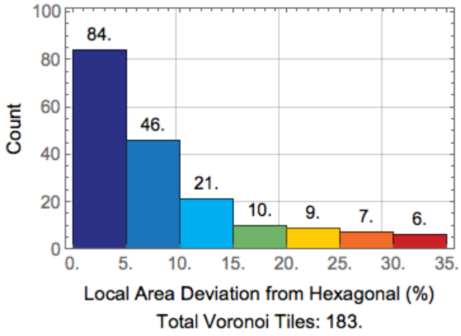

}}

```
(*  Overlay the Nanoparticle Images with the Voronoi Tessellation:  *)
(* a) Image Dimensions tells how many pixels the image is  *)
ImageDimensions[images[[1]]]

(* b) Rescale the points by:  image/box  size  *)
vor = PlotVoronoiTessellations[(410 / 1000) * xylene2000][[1]];

(* c) The double ImageReflect is needed
  because the jpeg image {0,0} starts at the top left... *)
(*     ...while xy data starts in the bottom left as the origin.  *)
ImageReflect@Show[{ImageReflect[images[[1]]], vor }]
```

{410, 410}

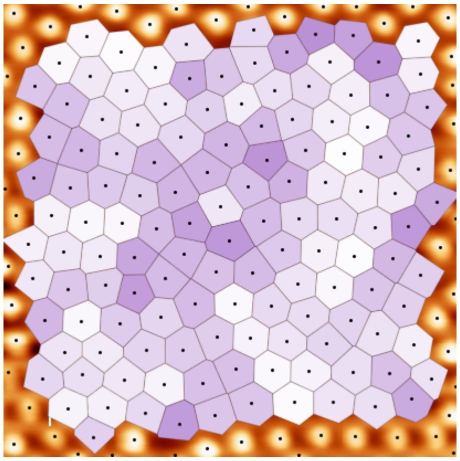

```
(* Scale Bars can be called individually *)
ScaleBars[#] & /@ {"voronoi", "bop", "coord"}
```

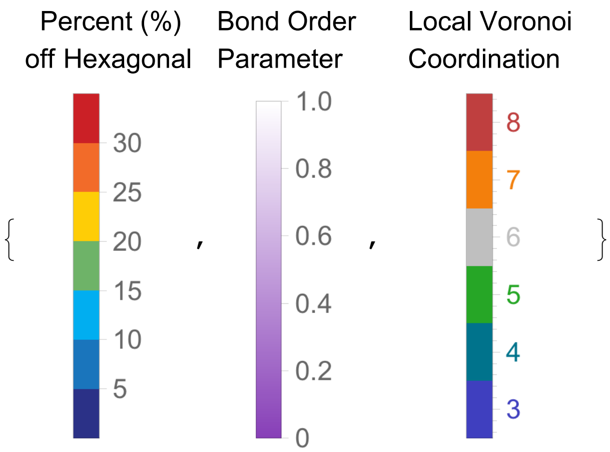

Pair Correlation Function - Distance between Particles

```
? PairCorrelationFunction
```

"PairCorrelationFunction[data2d , OptionsPattern[]]=Given a set of data2d={{x_1,y_1}...{x_n,y_n}}, returns a table {g(r),r} from r=0.0 to r=(maxBins * binSpacing). This is an ensemble definition. Multiple data2d={{$conf_1$}...{$conf_n$}} with the same density can be input and will be averaged.\n\nOptionsPattern=binSpacing→1,maxBins→1,box→{},edge→ToString[trunc],lattice→ToString[none], hexNormalized→False.\nSelect edge corrections (edge→ ⌗): ToString[pbc] || ToString[image] || ToString[noedge]\nChoosing lattice→ToString[hex] will Apply the proper Periodic Boundaries when using HexagonalLattice[periodic → True]\n"

```
(*  Basic Plot for the Pair Correlation Function                *)
(*  Returns the Plot of the first-
 peak normalized g(r) thats Smoothed + Hexatic Lattice *)
(* May take some time to complete:  (1-2 mins for large data sets) *)

PlotPairCorrelationFunction[xylene2000, names → nameList[[1]]]
```

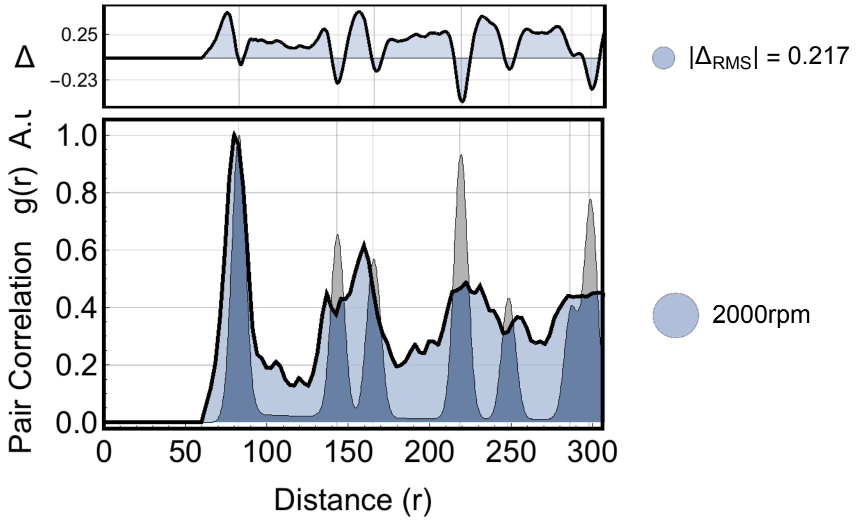

	First Peak g(r)	Expected Spacing (Hexatic Lattice)	Expected Disorder (Hexatic Lattice)	Particles (data)	Parti (latt
2000rpm	79.9868	82.6236	2.5267	183	187

```
(* Additional Option #1 - normalized *)
(* Remove the normalization of the
 first peak to see the Absolute Value of g(r)  *)

PlotPairCorrelationFunction[xylene2000,
 normalized → False, names → nameList[[1]]]
```

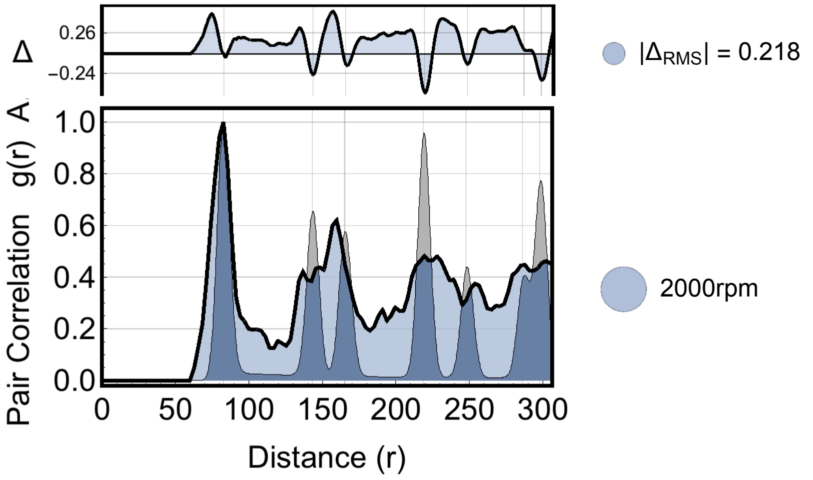

	First Peak g(r)	Expected Spacing (Hexatic Lattice)	Expected Disorder (Hexatic Lattice)	Particles (data)	Parti (latt
2000rpm	82.6931	82.6236	2.5267	183	187

```
(*     Additional Option #2 - lattice                              *)
(*     Normalizes the x-axis to the expected diameter              *)
(*     Can choose the Reference Lattice:  "hex"  or "square"      *)
PlotPairCorrelationFunction[xylene2000, names → nameList[[1]], lattice → #] & /@
 {"hex", "square"}
```

{

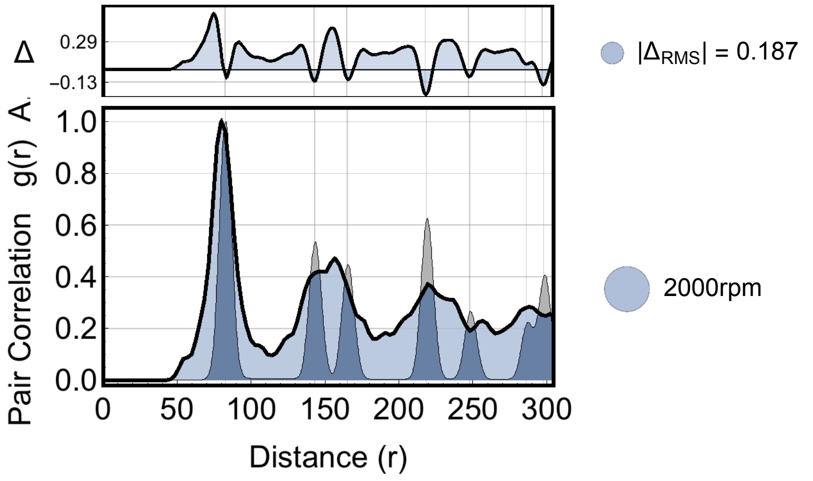

	First Peak g(r)	Expected Spacing (Hexatic Lattice)	Expected Disorder (Hexatic Lattice)	Particles (data)	Part (lat
2000rpm	79.9868	82.6236	2.5267	183	187

```
(*     Additional Option #3 - shells                              *)
(*     Vary the plotting distance with this parameter             *)
(*     Default Value = 4 shells                                   *)

(*    WARNING: If you ever get a divide by zero error (1/0)...     *)
(*     ... the first thing you should do is lower this value!!     *)
(*     Long range distances are bad for sparse data (N<200)        *)

PlotPairCorrelationFunction[xylene2000, names → nameList[[1]], shells → #] & /@
 {2, 3}
```

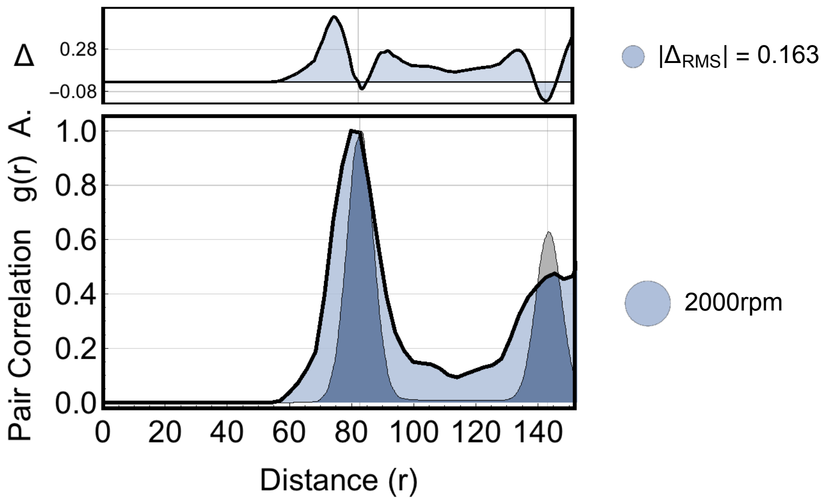

	First Peak g(r)	Expected Spacing (Hexatic Lattice)	Expected Disorder (Hexatic Lattice)	Particles (data)	Part (lat
2000rpm	79.8364	82.6236	2.5267	183	187

```
(*     Additional Option #4 - boots                                   *)
(*     Determines the Number of Randomized Displacements used to Smooth *)
(*     Default is 25                                                  *)
PlotPairCorrelationFunction[xylene2000, names → nameList[[1]], boots → #] & /@
 {1, 200}
```

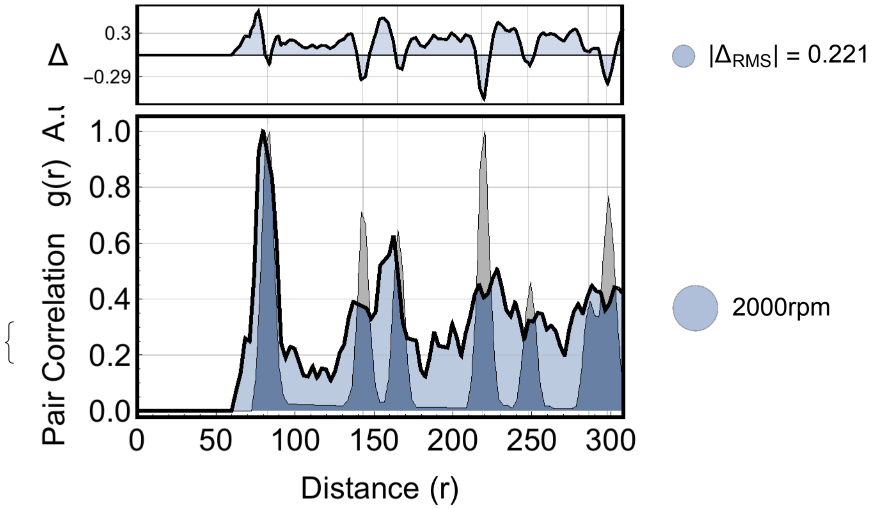

	First Peak g(r)	Expected Spacing (Hexatic Lattice)	Expected Disorder (Hexatic Lattice)	Particles (data)	Part (lat
2000rpm	79.6861	82.6236	2.5267	183	187

```
(*     Additional Option #5 - xLabel                  *)
(*     Change the x-axis label to have any string.    *)
PlotPairCorrelationFunction[xylene2000,
 names → nameList[[1]], xLabel → "------>  Changed Label (nm)"]
```

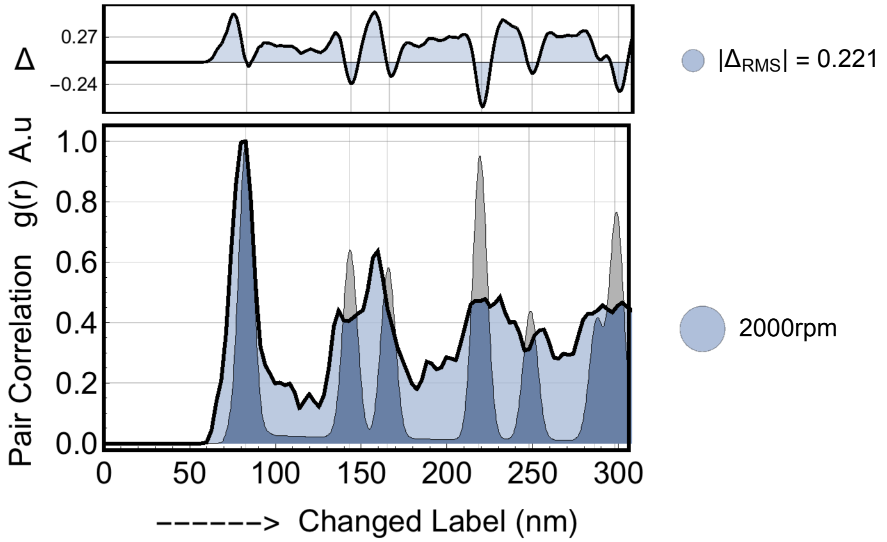

	First Peak g(r)	Expected Spacing (Hexatic Lattice)	Expected Disorder (Hexatic Lattice)	Particles (data)	Parti (latt
2000rpm	82.3924	82.6236	2.5267	183	187

```
(*  Call in a List of Data:  *)
(* - Automatically detects multiple {x,y} data and plots it as a stack.  *)
(* - Residual functions at the top are Hexatic Lattice Substractions. *)
(* - All Options above work with this stacked version as well.  *)

PlotPairCorrelationFunction[xyXyleneList, names → nameList]
```

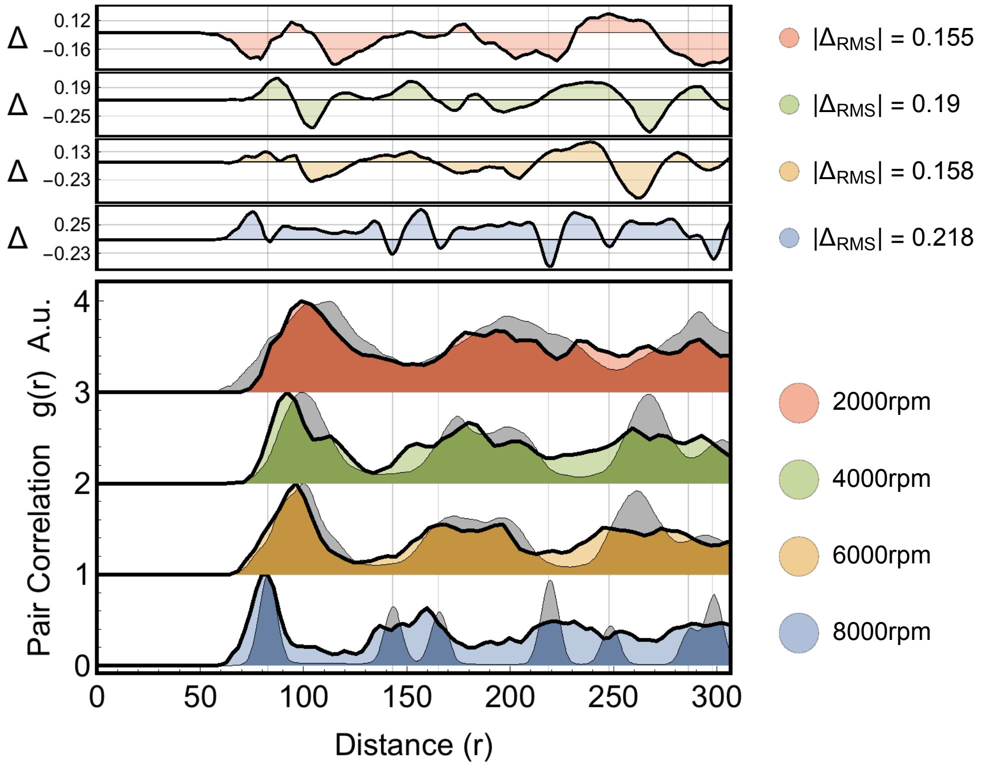

	First Peak g(r)	Expected Spacing (Hexatic Lattice)	Expected Disorder (Hexatic Lattice)	Particles (data)	Parti (latt
2000rpm	79.9868	82.6236	2.5267	183	187
4000rpm	96.3248	97.0426	7.45882	131	136
6000rpm	91.9299	100.223	6.63668	126	130
8000rpm	99.0041	107.439	11.4126	107	114

In[135]:=

```
(*    MAP in a List of Data:    *)
(* Returns the Individual Plots for each Configuration in the List *)

(*    Note the:  "/@"  NOT  "@"      *)

PlotPairCorrelationFunction /@ xyXyleneList
```

{

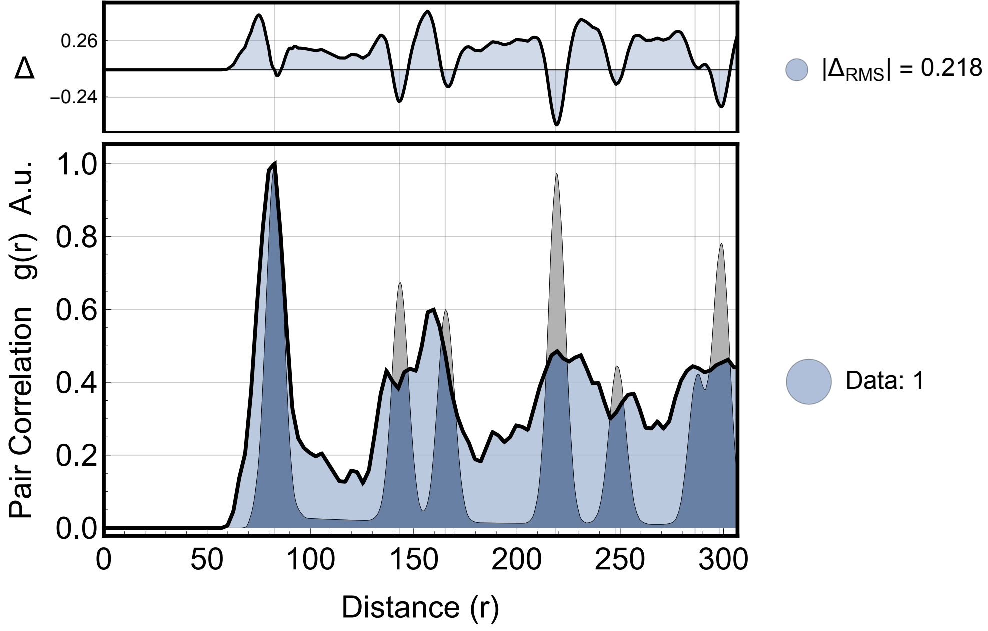

,

	First Peak g(r)	Expected Spacing (Hexatic Lattice)	Expected Disorder (Hexatic Lattice)	Particles (data)	Particles (lattice)
Data: 1	82.6931	82.6236	2.5267	183	187

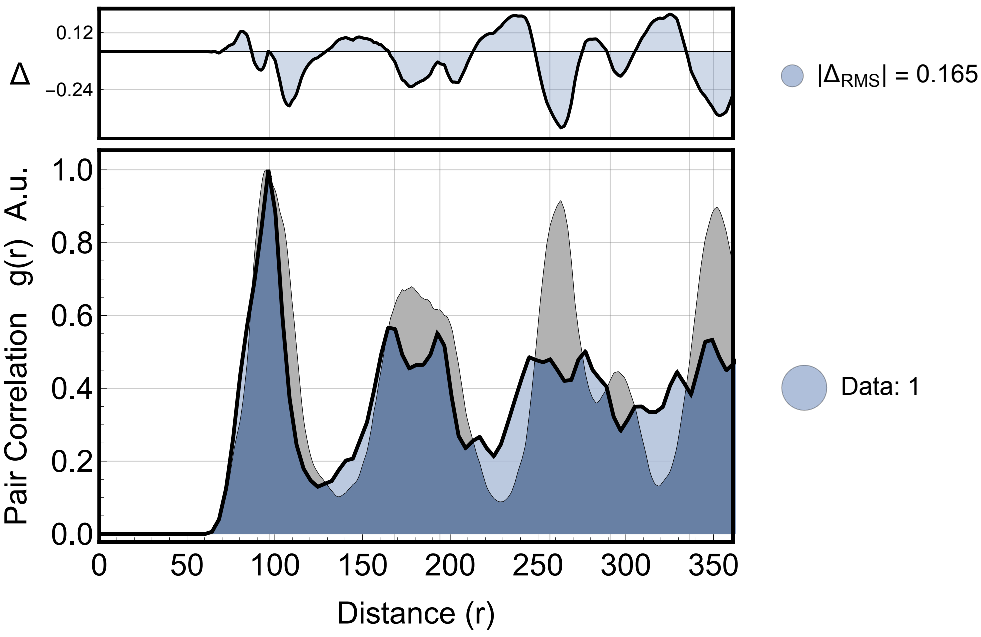

,

	First Peak g(r)	Expected Spacing (Hexatic Lattice)	Expected Disorder (Hexatic Lattice)	Particles (data)	Particles (lattice)
Data: 1	96.3248	97.0426	7.45882	131	136

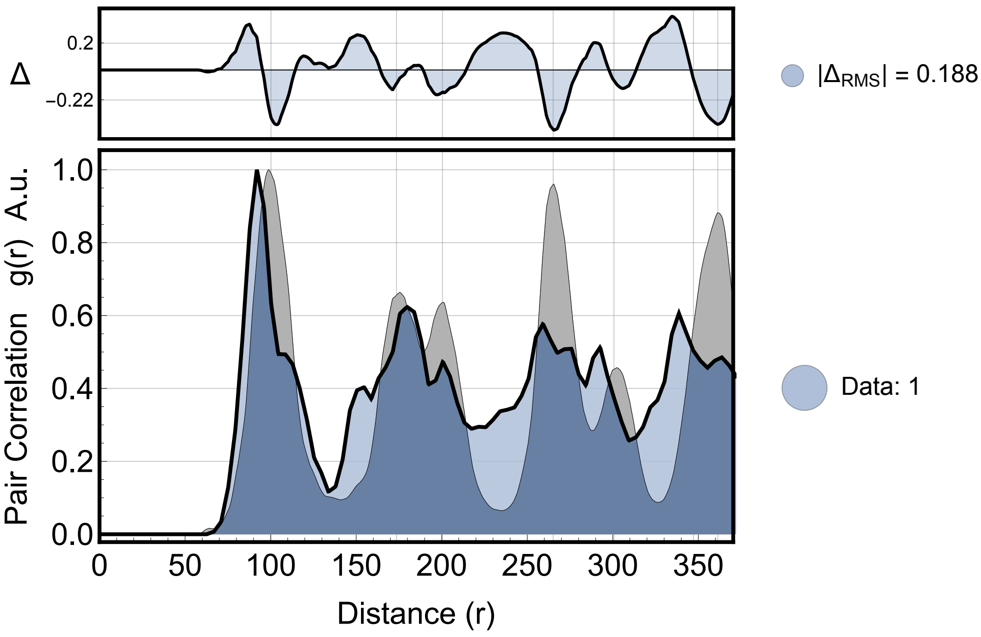

,

	First Peak g(r)	Expected Spacing (Hexatic Lattice)	Expected Disorder (Hexatic Lattice)	Particles (data)	Particles (lattice)
Data: 1	91.9299	100.223	6.63668	126	130

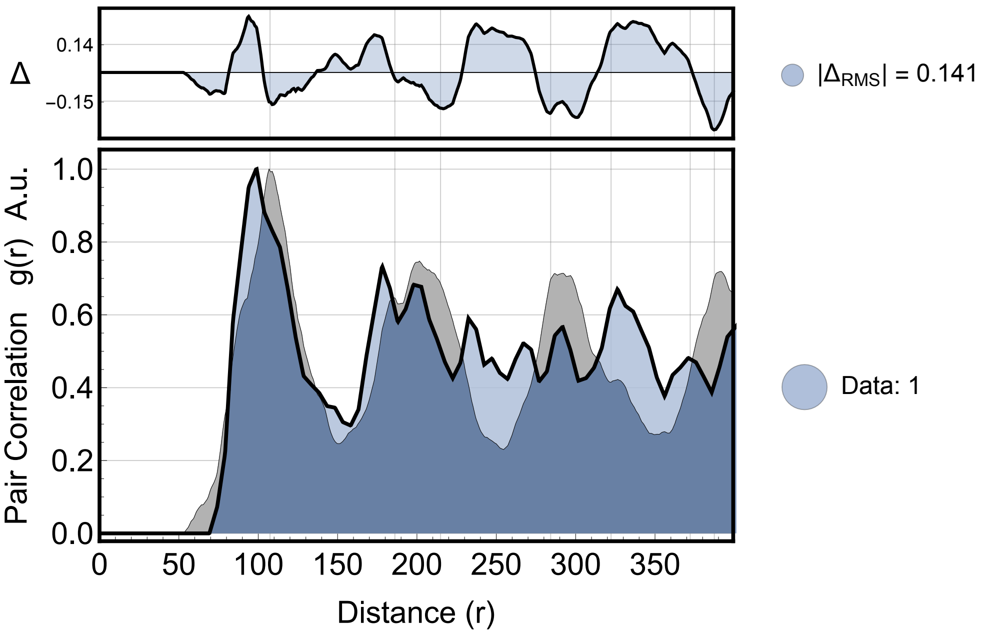

}

	First Peak g(r)	Expected Spacing (Hexatic Lattice)	Expected Disorder (Hexatic Lattice)	Particles (data)	Particles (lattice)
Data: 1	99.0041	107.439	11.4126	107	114

```
Export["PCFs.pdf", %];
```

```
(*    Distance Estimation from: Mathematica's Nearest[] Function   *)

AverageFirstNeighbourDistance /@ xyXyleneList
```

{71.3071, 82.635, 85.4448, 89.7071}

```
(*    Distance Estimation from: First Peak of g(r) - (no smoothing here)  *)

FirstPeakValue /@ (PairCorrelationFunction[#] & /@ xyXyleneList)
```

{82.8435, 96.5914, 96.1881, 103.78}

```
(*    Distance Estimation from: Voronoi Areas                        *)
(*    Returns:  { hexatic spacing,  lattice disorder parameter }   *)

ExpectedHexagonalDiameter /@ xyXyleneList
```

{{82.6236, 2.5267}, {97.0426, 7.45882}, {100.223, 6.63668}, {107.439, 11.4126}}

```
? PlotPcfDifference
```

"PlotPcfDifference[PairCorrelationList , OptionsPattern[]]=Difference Spectrum of PairCorrelationFunction[]. The values for: ref={g(r),r} and func={g(r),r} need to be lists.\nOptionsPattern[]=(xmax→0,normalized→True,shapes→{},stacked→False,names→{},xLabel→{},refIndex→1)\n\n xmax → 1 : Cut-off distance for g(r) where r_{max}=xmax.\n\nnormalized → True : Automatic rescalling of the x-axis to\n\nshapes → {} : List of Polygons to be used as the Legend Markers where - shapes→{$polygon_1$, ... , $polygon_n$} \n\nstacked → False : Option to overlay curves, or to stack them with an offset. If stacked→True, it will produce the stacked version. Multiple PairCorrelationFunction[] can be set with: func={$g(r)_1$, ..., $g(r)_n$};\n\nxLabel → {} : String used to label the distance axis. If xLabel→ToString[Normalized Diameter], the function changes the gridlines to the expected positions of the hexagonal lattice.\n\nrefIndex → 1 : Defines which PairCorrelation[] in the list is chosen as the reference for all others to be subtracted from. "

```
(*    Allows a comparison of any collection of graphs  *)
(*    Residual functions at the top
 are substractions relative to first data set.*)


PlotPcfDifference@(PairCorrelationFunction /@ xyXyleneList)
```

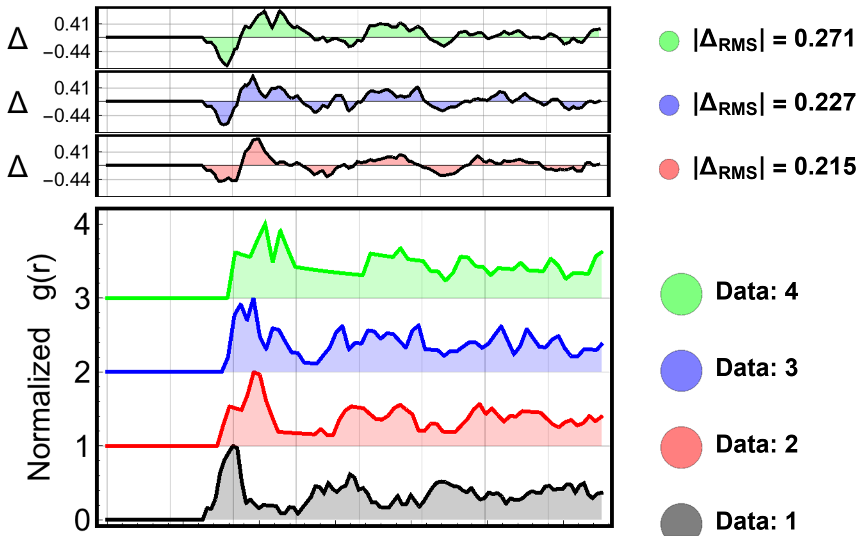

Bond Order - Angular Orientation of Neighbours

```
? BondOrderParameter
```

BondOrderParameter[unBoundedData2D , whichL , OptionsPattern[]]= Calculates the Bond Order using Delaunay triangulation for neighbour definition. Defaults are to remove edge particles and apply weighting of (voronoiBondFacet/totalVoronoiPerimeter) to every bond.

OptionsPattern=passVor→{},fast→True,edge–>ToString[NoEdge],vorbop→True,box→{},normalized→False,debug→False.

```
(*  Extract the local bond order parameter for each particle *)
(*  Call using: the data and symmetry number   *)

bop = BondOrderParameter[xylene2000, 6];
Mean[bop]
StandardDeviation[bop]
```

```
0.770507

0.142593
```

```
? CoordinationNeighbourCloud
```

```
(* Coordination cloud shows the probability
 of Neighbours relative to each object *)
(* Similar to the Fourier Transform,
except it can be separated by Coordination   *)

(CoordinationNeighbourCloud /@ xyXyleneList)[[All, 2]]
```

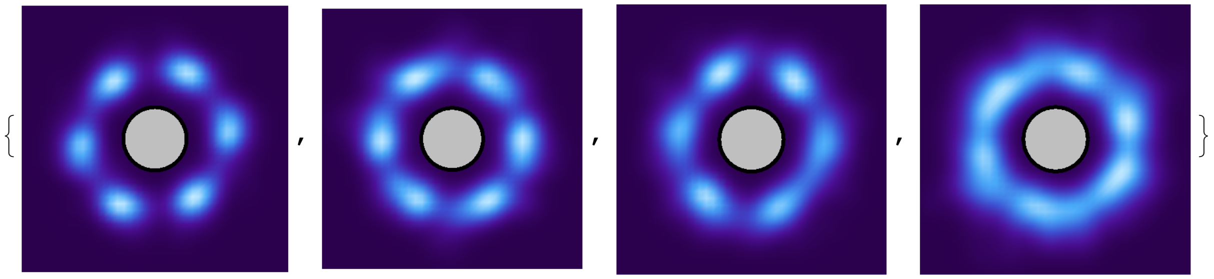

```
(*   Only select the particles that have 7 neighbours *)

CoordinationNeighbourCloud[xylene2000, coord → 7]
```

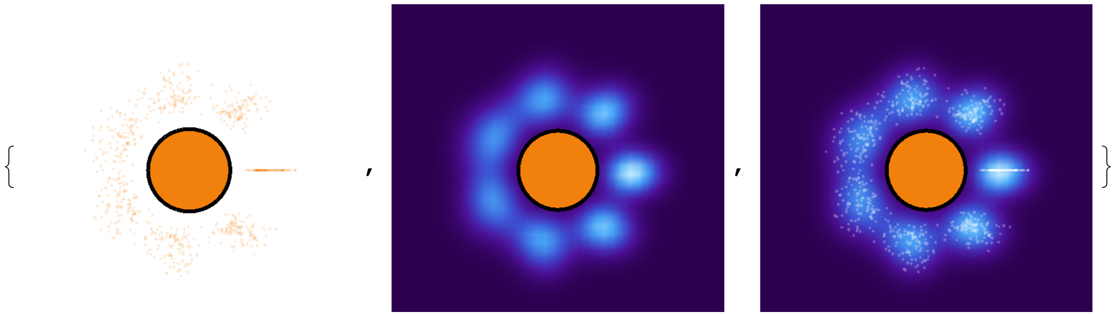

Diffraction - Correlation in Inverse Space

```
? DiffractionPatternFromPosition
```

```
(*  Plots the Diffraction (Fourier Transform) using a combination of:  *)
(*   ...Mathematica's ImagePeriodogram and ADO FFT (below)  *)

GraphicsRow[DiffractionPatternFromPosition /@ xyXyleneList, ImageSize → Large]
```

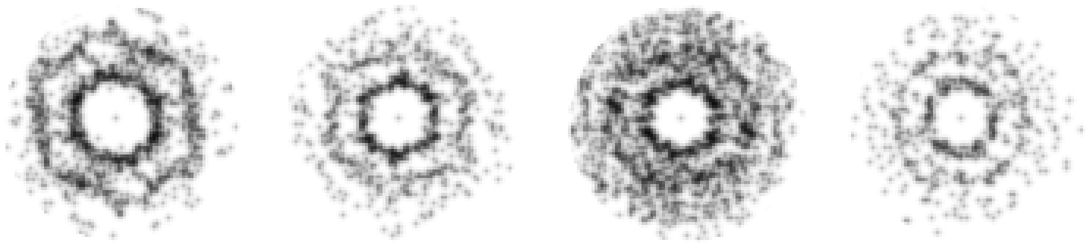

```
(*  Programmed FFT  *)

GraphicsRow[DiffractionPatternFromPosition[#, annulus → False] & /@ xyXyleneList,
 ImageSize → Large]
```

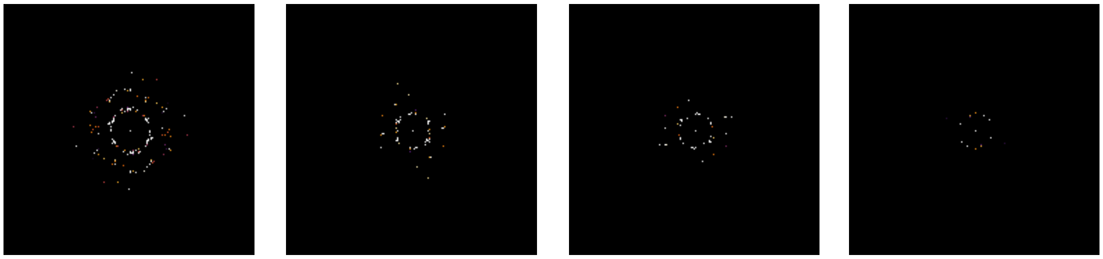

Supplement: Supplementary file 1 — Supporting Information [file 41598_2017_18894_MOESM1_ESM.pdf]
